# Music of infant-directed singing entrains infants’ social visual behavior

**DOI:** 10.1073/pnas.2116967119

**Published:** 2022-11-02

**Authors:** Miriam D. Lense, Sarah Shultz, Corine Astésano, Warren Jones

**Affiliations:** ^a^Department of Otolaryngology - Head and Neck Surgery, Vanderbilt University Medical Center, Nashville, TN 37232;; ^b^Vanderbilt Kennedy Center, Vanderbilt Brain Institute, Vanderbilt University, Nashville, TN 37232;; ^c^Marcus Autism Center, Children’s Healthcare of Atlanta, Atlanta, GA 30329;; ^d^Division of Autism & Related Disabilities, Department of Pediatrics, Emory University School of Medicine, Atlanta, GA 30307;; ^e^Laboratoire de NeuroPsychoLinguistique, Université Toulouse Jean Jaurès, 31058 Toulouse, France;; ^f^UMR 5267 Praxiling, Université Paul Valéry, 34000 Montpellier, France;; ^g^Center for Translational Social Neuroscience, Emory University, Atlanta, GA 30307

**Keywords:** infant-directed singing, rhythm, entrainment, social engagement, visual attention

## Abstract

Infant-directed singing is a culturally universal musical phenomenon known to promote the bonding of infants and caregivers. Entrainment is a widely observed physical phenomenon by which diverse physical systems adjust rhythmic activity through interaction. Here we show that the simple act of infant-directed singing entrains infant social visual behavior on subsecond timescales, increasing infants’ looking to the eyes of a singing caregiver: as early as 2 months of age, and doubling in strength by 6 months, infants synchronize their eye-looking to the rhythm of infant-directed singing. Rhythmic entrainment also structures caregivers’ own cueing, enhancing their visual display of social-communicative content: caregivers increase wide-eyed positive affect, reduce neutral facial affect, reduce eye motion, and reduce blinking, all in time with the rhythm of their singing and aligned in time with moments when infants increase their eye-looking. In addition, if the rhythm of infant-directed singing is experimentally disrupted—reducing its predictability—then infants’ time-locked eye-looking is also disrupted. These results reveal generic processes of entrainment as a fundamental coupling mechanism by which the rhythm of infant-directed singing attunes infants to precisely timed social-communicative content and supports social learning and development.

## Significance

Singing to infants is observed in all human cultures. Beyond known roles in infant soothing and social bonding, this study shows that singing to infants elicits physical entrainment, an elemental phenomenon enabling synchronization of wide-ranging physical and biological processes. Here, singing synchronizes subsecond social visual interactions between infants and adults. When an adult sings expressively to an infant, infants often increase their looking to the adult’s eyes around the musical beats. Adults also synchronize their facial expressions in time to their singing, presenting facial expressions on the beats that are more positive and engaging than those between beats. The rhythm of infant-directed singing thus entrains rich social-communicative interpersonal engagement, providing a remarkable yet ready-made means of supporting infants’ social development.

Music’s role in social bonding begins in infancy, with caregivers singing to their infants to soothe, placate, and engage them (1). This phenomenon, infant-directed singing, is culturally universal (2, 3) and promotes affiliative bonding (4). Infant-directed singing captures infants’ attention (5, 6), modulates infants’ arousal (5, 7–9), and reduces infants’ distress (8, 10) [even more so than infant-directed speech (8, 10)]. For caregivers, singing to infants regulates their own arousal levels (9, 11) and increases their perceived affective connection to their infants (11–13).

Infant-directed singing’s outward simplicity and universality belies a nonetheless complex and highly multimodal phenomenon: Infant-directed singing presents a wide range of acoustic cues differently from either speech or noninfant-directed singing, with greater amplitude and range in amplitude, increased and more variable pitch, reduced tempo, and prolonged relative duration of stressed to unstressed syllables (4, 14, 15). These exaggerated acoustic cues are also accompanied by a wider range of nonacoustic visual information: when singing to infants, integrated with their vocal acoustic cues, caregivers present more positive emotional facial expressions as well as increased communicative gestures and physical movements (moving head, hands, body, as well as bouncing or patting the infant) (6, 16).

One reason that infant-directed singing may be a powerful medium and force for social bonding is that it presents rich social-communicative information within a rhythmic structure that is highly predictable and temporally salient. The rhythm of infant-directed singing is structured by regular, predictable beats with heightened contrast between alternating strong and weak beats (metrical structure) (15–17). In adults, predictable beat-based rhythms enable individuals to plan and coordinate time-aligned behaviors, and that coordination, in turn, increases social affiliation (18, 19). In infancy, even passive experience of interpersonal rhythmic coordination increases infants’ subsequent social preferences and helping behaviors (20, 21).

Considering the mechanism by which infant-directed singing may facilitate social behaviors, the process of physical entrainment presents a compelling candidate. Entrainment is known to enable interaction across a broad range of phenomena, from mechanical and electrochemical coupling (22), to phase-locking of cells in a network (23), to the synchronization of animals’ activity (24, 25). In the first months of life, despite infants’ rapidly developing but still quite immature attentional, communicative, and motoric capacities (26), processes of entrainment may enable infants and caregivers to attune their interactions.

In the present study, to investigate whether the rhythm of infant-directed singing entrains infant social adaptive action, we measured whether infants’ visual attention varied in time with the rhythm of infant-directed singing. Specifically, we measured the extent to which infants’ looking to caregivers’ eyes was or was not modulated in time with the rhythm of infant-directed singing. We focused our investigation on infants’ attention to eyes because eye-looking is an early-emerging (27–29) and highly conserved (30, 31) mechanism of social adaptive action. Attention to others’ eyes supports healthy social communication and development (27, 32, 33) and increases “neural coupling” between interacting partners (32). Our primary hypothesis was that if rhythmic entrainment to infant-directed singing scaffolds social adaptive action, then infants’ looking to caregiver’s eyes should synchronize with that rhythm. We also hypothesized that rhythmic entrainment should synchronize caregivers’ own behavior and their deployment of redundant social cueing.

We tested our primary hypothesis in infants at 2 and 6 mo of age. At 2 mo, infants can remember and discriminate rhythms and melodies (34, 35), and they also first show clear evidence of actively engaging with others in a socially contingent and interactive manner (36): they coo in response to faces (37), begin to use eye contact contingently (27, 33), and smile reciprocally in relation to caregiver vocalization and affective state (37). Later, by the age of 6 mo, infants are already highly experienced in musical games (38) and face-to-face play (39), and they increasingly produce their own rhythmic behaviors [such as rhythmic babbling and hand movements (40)].

**Fig. 1.**
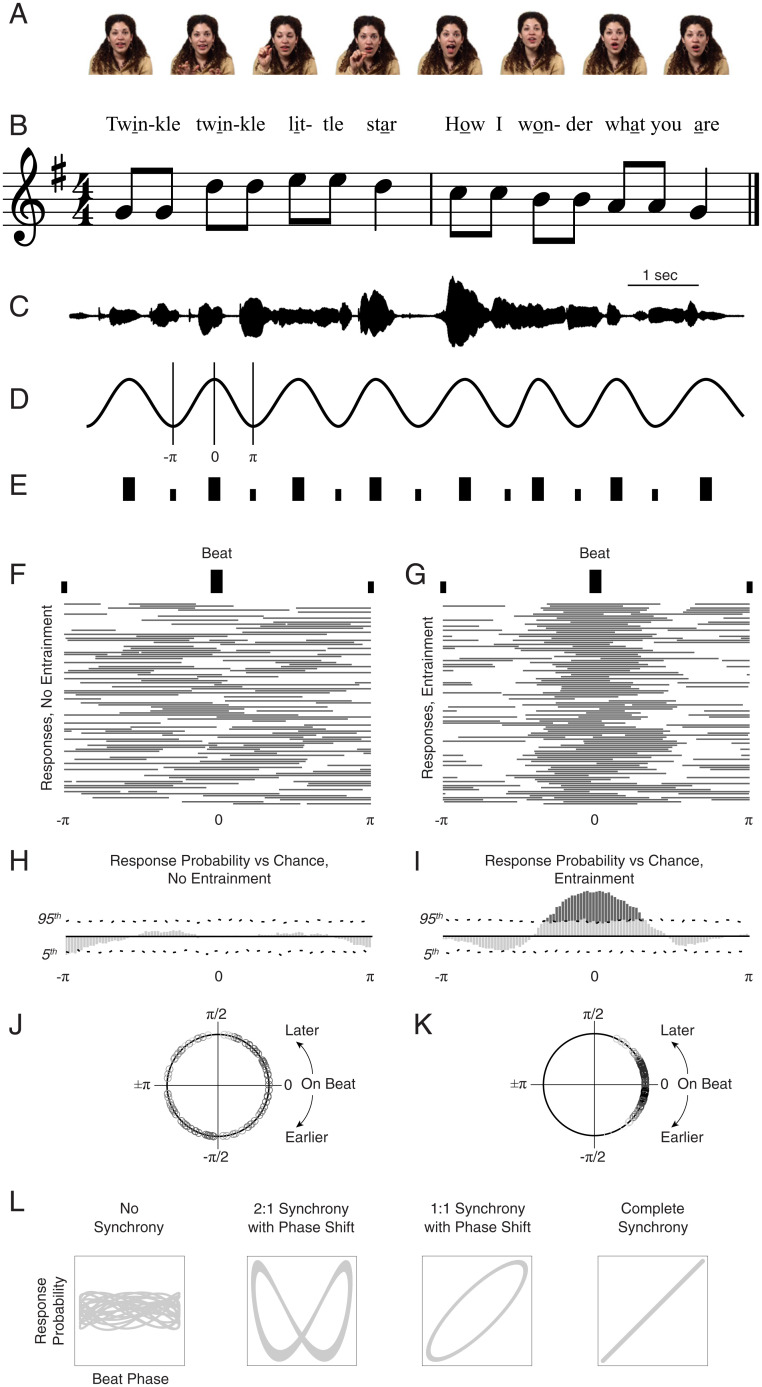
Quantifying the effects of rhythmic entrainment in infant-directed singing. (*A*) Still images from audio–video recordings of infant-directed singing. (*B*) Lyrics and musical notation for the first two phrases of “Twinkle, Twinkle, Little Star.” (*C*) Audio amplitude waveform for the song phrases in *B* sung by the actress pictured in *A*. (*D*) Schematic sine wave with peaks aligned to the beat of the audio as sung. One cycle (−π to +π) is marked by vertical lines, with the beat centered. Note that the interbeat interval, true to natural singing, varies and is not perfectly isochronous. (*E*) Schematic beat markings for strong beats (large black bars) and weak beats (small black bars). (*F* and *G*) Raster plots of archetypal response data that are either unaligned (*F*) or aligned (*G*) in time with the beat of infant-directed singing. Horizontal lines in each raster plot represent response events (hypothetical looking response when a line is plotted, not looking when not); each row corresponds to a single beat cycle. (*H* and *I*) Peristimulus time histograms summarizing raster data for archetypal responses that are either unaligned (*H*) or aligned (*I*) in time with the beat. Dotted horizontal lines are 95% confidence intervals for response variation expected by chance alone (one-sided); solid horizontal line is median expected by chance. (*J* and *K*) Phase portraits of archetypal responses either unaligned (*J*) or aligned (*K*) in time with the beat; each circle represents the phase of an individual’s looking response at the timing of the beat. (*L*) Lissajous curves also quantify synchrony between two time-varying signals: from no synchrony (as would be expected for data in *F*, *H*, and *J*), to higher-order synchrony with phase shift (here, two periods of output signal correspond to one period of modulating signal), to 1:1 phase synchrony (synchronized with 1:1 periods but with phase shift in timing), and complete synchrony (1:1 synchrony with 0 phase shift, as would be expected for data in *G*, *I*, and *K*).

We presented infant-directed singing to independent samples of 56 2 mo olds and 56 6 mo olds (Fig. 1 *A* and *B*) [mean(SD) age = 2.7(0.46) mo, range 1.7 to 3.4, 43% male, versus 6.2(0.38) mo, range 5.5 to 7.4, 57% male]. We used audiovisual recordings of infant-directed singing to create an explicit, unidirectional test of infant entrainment. While coordination of actual infant–caregiver interaction is, of course, bidirectional (41, 42), in our experimental design, infant behavior could have no effect on caregivers (the audiovisual recordings); if the two became synchronized, the effect would necessarily be due to infant entrainment to caregiver cueing (rather than caregiver accommodation). (For specifics on use of entrainment terminology in this report, see *SI Appendix*.)

Nursery rhymes (such as “Twinkle, Twinkle Little Star” and “Old MacDonald”) were sung by nonprofessional singers, with naturally occurring variation in tempo, amplitude, and pitch (quantified and described in greater detail in *SI Appendix*[Sec s7]). Nine audiovisual recordings were used, with audio sampled at 44.1 kHz and video at 30 frames per second. Each recording was ∼24 s in duration (range: 18.2 to 29.4 s). The rhythmic structure of caregiver singing (Fig. 1 *C*–*E*) was quantified by coding vowel durations of all metrically strong syllables within each song (as underlined in “Twinkle twinkle little star…”), referred to herein as “beats” for brevity. Coding was accomplished by visualization of each speech waveform and spectrogram, as well as by interactive playback (*SI Appendix*[Sec s7]) (15, 43, 44). In total, consistent with song notations, 227 beats were presented across 9 recordings. An advantage of this experimental design is that a high number of beat trials are presented within a short absolute duration (appropriate for infant testing).

For each singing caregiver, eye regions were bitmapped in each frame of video (*SI Appendix*, Fig. S1 *A* and *B*). Infants’ visual scanning was measured via eye-tracking equipment (ISCAN), and peristimulus time histograms (PSTHs) were used to examine whether mean levels of eye-looking were modulated in time to the rhythmic structure of caregiver singing (paradigmatic examples of nonmodulation and modulation are given in Fig. 1 *F*–*I*). We also examined the timing of individual synchronized responses via phase plots and Lissajous curves (paradigmatic examples given in Fig. 1 *J*–*L*). Please see *SI Appendix* for full description; through these analyses, we investigated the behavior of human infants within a mathematical framework that is common to other studies of elemental entrainment processes (22–25).

In brief, we analyzed whether infant eye-looking synchronizes to the rhythm of infant-directed singing. For each age group, we measured timing of eye fixations relative to the timing of metrically strong beats within the singing. For comparison, we also measured timing of fixations relative to the timing of high fundamental frequency (proxy for perceived pitch) and high root-mean-square amplitude (proxy for perceived loudness), given prior evidence that these acoustic cues attract infants’ attention (45, 46). Effects were quantified using PSTHs (47), measuring fixations to the eyes in 33.3-ms bins (one bin per video frame) in a window spanning 433.3 ms before and after each event (beat, high frequency, or high amplitude). Window width was selected to match the average interonset interval between all metrically strong beats across all videos (approximating one period; meter refers to the hierarchical organization of alternating strong and weak beats). We used permutation testing to determine whether observed results (actual modulation in timing of eye-looking relative to each stimulus event type) differed from results expected by chance for each age group (48). For between age-group comparisons of PSTH magnitude and shape, we also used permutation testing. See *SI Appendix* for full description.

## Results

### Control Analyses.

Given infant participants’ young ages, we first confirmed that task completion was adequate for further analyses (*SI Appendix*, Fig. S1 *C*–*J*). Average calibration accuracy was within 1° of visual angle and did not differ significantly between age groups (*SI Appendix*, Fig. S1 *C*–*F*). At 2 and 6 mo, infants had similar amounts of usable data collected: 96.9(38.0) beat trials per child at 2 mo and 105.3(55.9) beat trials per child at 6 mo [*t*_110_ = 0.92, *P* = 0.36, data given as mean(SD)] (*SI Appendix*, Fig. S1*G*). Infants at both ages had similar proportions of overall time spent fixating [mean(SD) at 2 mo: 59.4%(16.4); 6 mo: 63.7%(13.5); *t*_110_ = 1.50, *P* = 0.14] (*SI Appendix*, Fig. S1*H*), as well as proportion of time spent fixating on the eyes [2 mo: 31.3%(22.6); 6 mo: 31.6%(19.2); *t*_110_ = 0.06, *P* = 0.95] (*SI Appendix*, Fig. S1*I*). There was no significant between-group difference in rate of fixations [2 mo: 2.34(0.83); 6 mo: 2.29(1.26); *t*_110_ = 0.91, *P* = 0.36] (*SI Appendix*, Fig. S1*J*). We also measured infants’ abilities to precisely saccade toward and fixate nonsocial, nonmusical rhythmic targets (flashing/rotating geometric shapes with cooccurring audio), measuring latency to first saccade after onset (*SI Appendix*, Fig. S1*K*) and duration of first fixation (*SI Appendix*, Fig. S1*L*) (two behaviors that could impact entrainment-related measures). While there was no difference in mean latency nor median fixation duration (*t*_107_ = 0.35, *P* = 0.73 saccade latency and *z* = 1.45 *P* = 0.146 fixation duration, Mann–Whitney *U* test), 2 mo olds were more variable in latency to first saccade (*F*_1, 107_ = 15.9, *P* < 0.0001; Levene’s test for equality of variance). As with other aspects of development at these young ages (26), this increased variability would be consistent with less precise timing of more immature motor skills in 2 mo olds. For the present purpose, to ask and interpretably answer whether behavioral entrainment is present, the collected data demonstrate sufficiency even at the very young age of 2 mo.

### Entrainment of Infant Eye-Looking During Infant-Directed Singing.

**Fig. 2.**
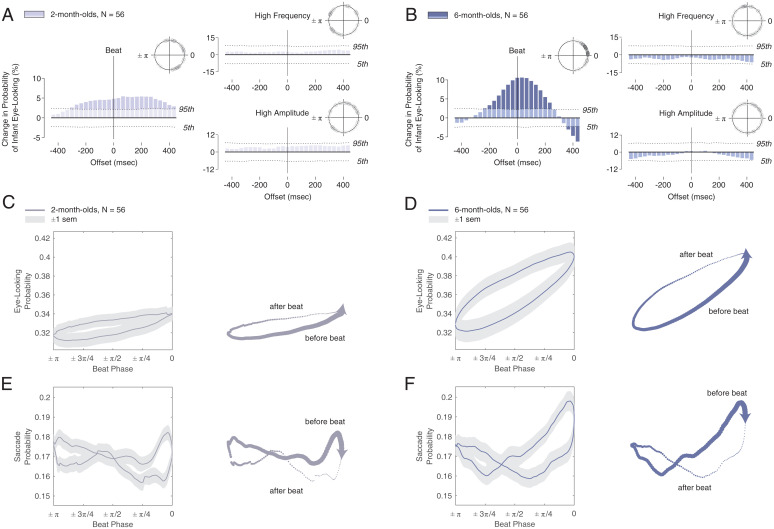
Infant eye-looking is time-locked to the rhythm of infant-directed singing. During infant-directed singing, we quantified the effects of beat, frequency, and amplitude on how 2-mo-old (*A*) and 6-mo-old (*B*) infants looked at the eyes of a singing adult. In both 2 mo olds (*A*) and 6 mo olds (*B*), infants significantly increased their looking to singers’ eyes, time-aligned to the beat of infant-directed singing. The observed increases in eye-looking time-locked to the beat were significantly greater in 6 mo olds (*B*) than 2 mo olds (*A*), quantified by permutation testing (*SI Appendix*[Sec s7]). At each age, there was no significant change in time-locked eye-looking relative to moments of either high frequency (*Upper Right*) or high amplitude (*Lower Right*). Dotted lines in all panels of *A* and *B* show 5th and 95th confidence intervals for change in eye-looking expected by chance (one-sided). Note that the scale of the ordinate differs across peristimulus time histograms: plots are scaled to align by probability of observed results (aligned at the 5th and 95th confidence intervals of results expected by chance). *Inset* phase plots show individual phase of eye-looking for all infants. Phase plots indicate that effects of the beat are observable not only at the group level (as shown in peristimulus histograms), but also in the individual behavior of a majority of infants at each age. (*C* and *D*) Lissajous curve for probability of infant eye-looking versus beat phase for (*C*) 2-mo-old and (*D*) 6-mo-old infants. Traces at the right of each panel show direction of Lissajous curve travel over time. In Lissajous curves, mean looking probability is plotted in blue while gray areas denote ±1 SEM. In all traces, the arrowhead denotes mean response level at the beat (beat phase = 0), with trace thickness denoting direction of travel (thickening as time moves forward, resetting immediately after the beat). (*E* and *F*) Probability of infant saccades versus beat phase for (*E*) 2-mo-old and (*F*) 6-mo-old infants. Probability of eye-looking in 6-mo-old infants shows 1:1 synchrony with ∼/5.5 phase shift. Saccades in 6 mo olds are synchronized at two saccade periods per one beat period, with maximum increase prior to (in anticipation of) the beat. For additional Lissajous analyses, see *SI Appendix*.

As seen in Fig. 2 *A* and *B*, at both 2 and 6 mo of age, time-locked to the rhythm of caregiver singing, infants increase their looking to the eyes at levels greater than expected by chance (*P* < 0.05). Moreover, beyond the aggregate effects plotted in PSTHs, we find that the phase of individual response is also remarkably time-aligned to the beat: within ±108 ms of the beat (±1/8 of one strong beat period), individual eye-looking reaches its local maximum value for 58.9% of 6 mo olds (33 of 56) and for 32.1% of 2 mo olds (18 of 56) (phase plots are given in *Upper Right Insets* of Fig. 2 *A* and *B*; each marker on the plot denotes the average phase response of one infant’s eye-looking at the songs’ strong beats). Both groups show significant time alignment: *V* = 33.03, *P* < 0.001 at 6 mo and *V* = 16.9, *P* < 0.001 at 2 mo [*V*-test for circular uniformity at the beat [ϕ = 0]). Complementary analyses conducted with Lissajous curves (Fig. 2 *C*–*F* and *SI Appendix*, *Supplementary Results* and Fig. S2) show phase synchrony between the beat sinusoid and continuously varying measures of eye-looking probability and saccade probability (with 1:1 and 2:1 phase synchrony, respectively, and with saccade probability increasing just prior to the beat) (Fig. 2 *C*–*F* and *SI Appendix*, Fig. S2).

Results also indicate a developmental progression (Fig. 2 *A*–*D*). While 2- and 6-mo-old infants both show eye-looking responses phase-locked to the beat, this increase in eye-looking had significantly tighter phase-locking (greater consistency of response around 0) at 6 mo (ϕ_var_ = 0.37) vs. 2 mo (ϕ_var_ = 0.70) (χ^2^ = 7.78, *P* = 0.005). This is similarly reflected in PSTH magnitude and shape: 2- and 6-mo-old infants both show significantly increased eye-looking time-locked to the rhythm of caregiver singing, however the magnitude of increased eye-looking was significantly greater for 6- versus 2-mo-old infants around 0 (between-group significant differences from −66.7 to 99.9 ms, *P* < 0.05, identified by permutation testing) (*SI Appendix*). In addition, in terms of magnitude of increase in eye-looking, within-group comparisons with levels expected by chance show an approximately twofold increase in looking at 2 mo, but a greater than fourfold increase at 6 mo. These differences may in part reflect motor maturation from 2 to 6 mo, and they support the notion that rhythmic entrainment of social adaptive action is observable already by 2 mo and increasingly robust by 6 mo.

Results observed for beats contrast with those observed for high frequency and high amplitude (Fig. 2 *A* and *B*). While moments of high frequency and high amplitude are both important prosodic markers of communicative emphasis (15, 49), and they both relate to the rhythm of the stimulus, they can also occur at times distinct from moments of rhythmic (metrically strong) importance; consequently, moments of high frequency and high amplitude provide related, but also at times dissociable, communicative cues. When analyzed as discrete potential drivers of infant eye-looking, neither high frequency nor high amplitude alone was sufficient to modulate infant eye-looking, in either 2- (Fig. 2*A*) or 6-mo-old infants (Fig. 2*B*) (“high” defined as values >90th percentile, tested at multiple other thresholds with consistent results) ([Sec s7]*SI Appendix*, *Supplementary Results*).

### Rhythmic Synchronization of Caregiver Cueing.

Observation of infants’ time-aligned looking to the rhythm of caregiver cueing raises important questions about what information caregivers present during metrically strong moments. Infant-directed communication is well known for exaggerated facial expressions, gestures, and movements (50, 51), but how those visual cues are organized in time to enhance information transfer is not well understood. Therefore, we investigated what visual information is also time-aligned with the beat. Specifically, we measured caregivers’ affective expression, facial motion, and markers of engagement relative to the rhythm of their singing. These singers were not professionals: any change in behavior occurred without training or overt instruction, and without explicit planning (they were merely asked to sing nursery rhymes to children).

**Fig. 3.**
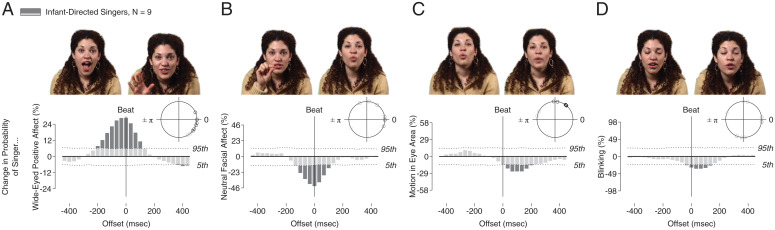
The rhythm of infant-directed singing is a structuring mechanism for infant-directed communication. Relative to each beat, we measured change in probability of caregiver displays of (*A*) wide-eyed positive affect, (*B*) neutral facial affect, (*C*) caregiver facial motion in the eye area, and (*D*) caregiver blinking. Time-locked to the beat of their own infant-directed singing, caregivers increase displays of wide-eyed positive affect, reduce neutral facial affect, reduce motion in the eye region, and reduce blinking [blink inhibition is a marker of engagement (31)]. Dotted lines show 5th and 95th confidence intervals for change expected by chance for each visual prosodic marker (one-sided); plots are scaled to align by probability of observed results. Images above plots are representative video stills for each analysis.

As seen in Fig. 3, time-locked to the rhythm of their singing, caregivers change the visual cueing they provide to onlooking infants. Time-aligned to metrically strong beats, caregivers significantly increase their displays of wide-eyed positive affect, with a maximum increase in probability of 28.8% above chance levels (Fig. 3*A*). Caregivers also significantly reduce displays of neutral facial expressions (43.4% decrease from levels expected by chance) (Fig. 3*B*) and they reduce motion in the eye area (25.9% decrease) (Fig. 3*C*). Finally, caregivers also synchronize their inhibition of blinking—a marker of visual attention and engagement (47)—in time with the rhythm of their singing (34.8% decrease) (Fig. 3*D*).

Across each of these metrics, caregivers reveal increased social-communicative cueing and engagement, unconsciously choreographed to the rhythm of their singing. This synchronization has the joint effect of creating a more engaging display for the onlooking infant (increasing probability of infant reaction) while also repeatedly and redundantly highlighting salient social information from the caregiver. In supplementary analyses, we analyzed how this confluence of cueing affects social information transfer developmentally, to support a child’s social adaptive learning (*SI Appendix*, *Supplementary Results* and Fig. S3): at 2 mo, entrainment is driven by the beat, with no entrainment to cooccurring presentation of wide-eyed, positive affect (*SI Appendix*, Fig. S3 *B* and *C*); at 6 mo, however, the timing of infant eye-looking is aligned with the beat but is also potentiated by a caregiver’s cooccurring affect (*SI Appendix*, Fig. S3 *E* and *F*). This developmental progression suggests that infant eye-looking becomes increasingly sensitive to added layers of social information, layers that are supported by the rhythmic structure of infant-directed communication. Note that while caregivers increase wide-eyed positive affect in time with the beat, they also present wide-eyed positive affect between beats, allowing us to contrast infant response (*SI Appendix*, Fig. S4): at neither 2 nor 6 mo of age do infants precisely time-align their eye-looking to wide-eyed positive affect irrespective of the rhythmic structure (this does not mean that infants do not look at wide-eyed positive affect [they do], only that the precise timing aligns with rhythmic structure rather than with the social-affective expression alone).

### Disrupting Rhythmic Predictability of Infant-Directed Singing.

As a final test of the extent to which changes in infant-looking depend upon the rhythmic structure of caregiver cueing, we experimentally manipulated that structure by varying rhythmic predictability. We resampled the original audiovisual recordings—which had naturally varying but predictable interbeat intervals—to reduce their predictability: in each song, two-thirds of the interbeat intervals were randomly varied by ±30% of their original duration, disrupting the original rhythmic structure and reducing beat-to-beat predictability. Note that the reduction in predictability also serves as a strong experimental control for effects of simple motion and caregiver visual cueing: the entire range of head and facial motion and all affective cues are presented, while only their relative temporal predictability is manipulated.

To test effects of this manipulation, we first replicated the initial findings of increased eye-looking time-locked to the beat of the original (predictable) infant-directed singing. As in the first cohort of infants, an independent replication cohort—*n* = 33 6 mo olds, mean(SD) age = 6.2(0.36) months, range 5.0 to 6.8, 52% male—increased their eye-looking in time to the rhythm of caregiver singing at levels greater than expected by chance (*SI Appendix*, Fig. S5) (ϕ_var_ = 0.50, *V* = 6.8421, *P* = 0.036).

**Fig. 4.**
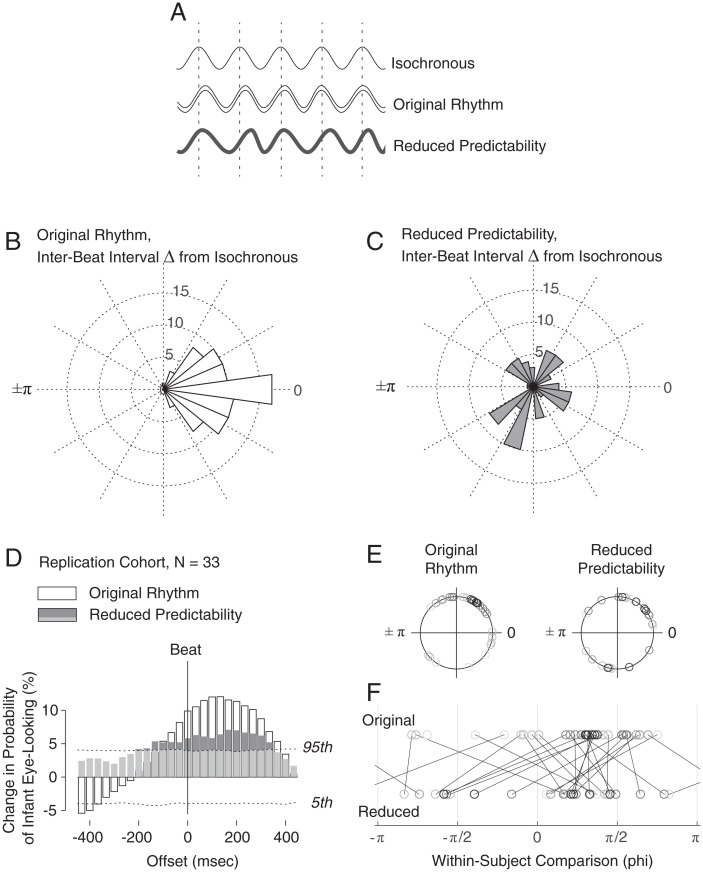
Reducing rhythmic predictability reduces synchronized infant eye-looking. (*A*) Infant-directed singing presents predictable rhythmic cueing with relatively regular interbeat intervals (although, as noted, interbeat intervals in natural singing are not perfectly isochronous). To test the effects of reducing rhythmic predictability on infants’ looking, we experimentally manipulated the rhythm of caregiver cueing. We resampled the original audiovisual recordings and introduced jittering to the interval timing: in each song, two-thirds of the interbeat intervals were randomly varied by ±30% of their original duration. In this way, jittering disrupted the original rhythmic structure and reduced beat-to-beat predictability (bottom waveform). (*B*) Polar histogram showing the extent of variation in timing of interbeat intervals in original recordings (difference, in interbeat intervals relative to isochronous signal). (*C*) Polar histogram showing the extent of variation in the reduced predictability condition, after experimental manipulation. (*D*) In a replication cohort of 6 mo olds (see also *SI Appendix*, Fig. S4 for replication results), time-locked eye-looking is reduced when infants view singing with reduced predictability. White bars show response to original, predictable singing (data are repeated in *SI Appendix*, Fig. S4*B* and compared to the discovery cohort 6-mo-old results). Gray bars show response to reduced predictability condition. Dotted lines show 5th and 95th confidence intervals for change in eye-looking expected by chance (one-sided). (*E*) Phase plots of eye-looking for individual infants in the original and reduced predictability conditions, and (*F*) within-subject comparisons of individual phase response across conditions: diagonal lines connect phase responses of individual infants across original rhythm (top row) and reduced predictability (bottom row) conditions; two infants had usable data in one condition but not the other and so have no connecting line.

However, when the replication cohort was presented with the experimentally manipulated, reduced predictability stimuli (Fig. 4 *A*–*C*), the reduction in cue predictability led to a reduction in entrained eye-looking: both time-alignment and magnitude of entrainment were reduced (Fig. 4 *D* and *E*). Response phase across infants was more variable (ϕ_var_ = 0.69, *V* = 5.3308, *P* = 0.08, trending toward but no longer significantly synchronized to the beat). Response magnitude was reduced at all time points of initially significant response: reductions ranged from 3.1 to 51.3% of the initial magnitude at each time point (e.g., at 133.3 ms after the beat; original response of 12.0% was reduced to 6.4%, a 46.8% reduction). Interestingly, some significant increase in eye-looking remains, an effect that may be due to infants’ ability to accommodate varying degrees of predictability.

## Discussion

Music’s facilitation of social behaviors is hypothesized to contribute to the evolution and continued importance of musical behaviors (52). The present results reveal that infant-directed singing, a culturally universal form of musical interaction, entrains infants’ gaze to caregiver’s eyes, providing a mechanism by which infant-directed singing supports social interaction on subsecond timescales and across development, observable as early as 2 mo and progressively strengthened by 6 mo. Time-aligned to the rhythm of infant-directed singing, the probability of infant saccades increases just prior to the beat, while the probability of infant eye-looking increases just after. This increased probability of infant behavior is optimally aligned with important changes in the caregiver: time-aligned to the rhythm of their own singing, caregivers unconsciously structure their actions, enhancing communicative value and cueing redundancy at each beat. In this way, rhythmic entrainment during infant-directed singing serves as a structuring mechanism for social adaptive action and information transfer, repeatedly and predictably delivering redundant cues to modulate attention at salient social moments, providing infants with key opportunities for social learning and supporting their developing sensitivity to layers of social-communicative meaning.

Early in development, infants’ sensory, motoric, communicative, and cognitive faculties are extremely immature, requiring extensive caregiver support to survive and modulate behavior (26). To optimally align infant abilities with those of a caregiver, it is incumbent upon caregivers to modulate and structure their own behavior to best support infant engagement and development. The present study shows that rhythm and rhythmic entrainment accomplish this, even for the caregivers’ own expressive visual cues (e.g., wide-eyed positive affect), which occur more robustly and maintain infant attention more fully during infant-directed singing than speech (6). Infant-directed singing can be seen as a quasiperformative context in which the caregiver intuitively uses exaggerated and expressive visual displays to successfully engage the infant. Trained performing singers in noninfant-directed contexts also use dynamic visual cues involving head, eyebrow, and lip movements to express emotions to an audience (53, 54). As with any performative interaction, the communicative relevance and impact of expressive cues are impacted by the performer, the audience, and the context in any given moment (55–57).

In the present study, by tracking moment-by-moment infant eye-looking, we find that infant-directed singing not only maintains infant attention in general but dynamically modulates it on subsecond timescales. As further evidence that rhythmic entrainment is the driver of these effects, infant-looking is not precisely time-aligned with other well-known and important visual and acoustic cues when response to those cues is analyzed irrespective of rhythmic structure. These results are consistent with rhythm as a temporal organizer of listeners’ experiences, not a contradiction with the importance of those features; rather, the results offer evidence that during infant-directed singing, rhythm organizes those and other features, and drives infants’ responses (*SI Appendix*, *Supplementary Results*).

Consistent with entrainment processes observed in other physical systems, here the rhythm of one system changes that of another, scaffolding the otherwise unconstrained behavior of 2 and 6 mo olds, to increase their eye-looking in time with moments when caregiver’s communicative cues carry greatest informational content. This shows that although what a caregiver expresses in unimodal cueing is important, when and how that cueing occurs may be more critical for the infant’s response and receipt of information. Rhythm (to specify the “when” of predictable repetition) and rhythmic entrainment (to specify the “how” of complementary redundancy) seem ideally suited to solving the task of supporting successful social information transfer between caregiver and child.

Critical to this process—and to the synchronized social engagement that results—is rhythmic predictability: disrupting the predictability of infant-directed singing also disrupts time-aligned eye-looking despite otherwise presenting the same complete range of acoustic and visual cues. Rhythmic predictability is, of course, a universal feature of music. Here we see that by supporting the synchronization of infants’ eye-looking and caregivers’ communicative-cueing, the rhythm of infant-directed singing may serve as an adaptive structuring mechanism to support infant-caregiver bonding and to enable infants’ developing social-communicative skills.

This is also notable in relation to earlier findings in the literature of nonsinging, free-play interactions: Cohn and Tronick (58) observed bouts of organized bidirectional influence between mothers and infants; the behaviors were autocorrelated over short intervals and cross-correlated with the preceding behaviors of each partner (stochastic and autoregressive in nature, rather than sustained periodic). In the present work, the rhythm of infant-directed singing creates a sustained periodic structure that organizes the caregiver’s behavior as well as the behavioral response of the infant. Infant-directed singing creates an opportunity to extend the bouts observed by Cohn and Tronick, and extend (through repetition) the predictability of the social information conveyed during those interactions. Infant-directed singing thus appears to take elements seen in spontaneous, stochastic cross-correlated behavior and make those elements periodic, predictable, and prolonged via musical rhythm.

Rhythmic entrainment may also provide a common mechanism to support other aspects of infant social development. In infant-directed speech, for example, caregivers also frequently increase rhythmicity through salient stress patterns (59, 60). More broadly, caregivers and infants coordinate the timing of their interpersonal behavior through turn-taking exchanges of gaze, vocalization, and gesture (61, 62) [with earlier success predicting more positive later outcomes in language, cognition, and attachment (63–65)]. One area of important future study concerns how changes in looking behavior relate more specifically to markers of attentional state (66), engagement (47), and learning. Recent work (66) has shown phasic pupillary response to musical rhythms, strongly predictive of deviants in a deviance detection task. In future work, the use of additional measures of dynamic attentional state and engagement, beyond fixation location, could index key elements of dynamic attending, perception and, ideally, give insight into predictive models for infant learning.

Notably, the rhythmicity and stereotyped exaggerations displayed by caregivers during infant-directed singing are also present in many (if not all) forms of infant-directed communication. Given their ubiquity, these behaviors are sometimes called “intuitive parenting”: no instruction is typically necessary because the behaviors emerge ontogenetically from the mutual adaptation of the infant–caregiver dyad and from the complementary matching of infant capacities and caregiver actions (table 1 in ref. 26). As infants’ skills mature, caregivers change their own behaviors to best support their child’s engagement in an interactive social feedback loop (including through their temporal structuring of speech and song) (67–72). The present results reveal rhythmic entrainment as a coupling mechanism that enables that matching and supports the emergence and maturation of those behaviors.

However, the processes that make entrainment possible also make it susceptible to disruption at multiple different links in the infant–caregiver dyad: sensitivity to social or rhythmic social information, accommodation of varying degrees of rhythmic predictability, and capacity to produce synchronized responses may all play important roles, beginning in early life, in the development of successful social interaction. Weaknesses at or among any one (or multiple) of these links may relate to difficulties in social interaction and communication, as seen in neurodevelopmental disabilities like autism. If an infant’s recognition, accommodation, or response is unsuccessful, disrupting infant–caregiver entrainment, this could have immediate effects on learning opportunities as well as on future interactions, with cascading consequences for development. More optimistically, quantifying and understanding the exact nature of any such disruptions may offer new avenues for effective and individualized intervention.

## Materials and Methods

A complete description of materials and methods can be found in the *SI Appendix*, *SI Materials and Methods*. Details on participants, stimuli, data acquisition and processing, and data analysis (including audio and visual stimuli quantification, peristimulus time histograms, phase analyses, and Lissajous curves) are provided. This research was based in the Marcus Autism Center, part of Children’s Healthcare of Atlanta and the Department of Pediatrics at Emory University School of Medicine. The study protocol was approved by the Institutional Review Board of Emory University School of Medicine (00060097, 00089562). Parents/legal guardians of all infant participants gave informed consent prior to participation.

## Supplementary Material

Supplementary File

## Data Availability

Anonymized data (eye-tracking and demographics) have been deposited in Open Science Framework at https://osf.io/gbjzw/ (73).
