# Supplementary material for: Music of infant-directed singing entrains infants’ social visual behavior
Source: Proc Natl Acad Sci U S A. 2022 Nov 2;119(45):e2116967119. doi: 10.1073/pnas.2116967119 (PMC9659341; doi:10.1073/pnas.2116967119)
Supplement: Supplementary File [file pnas.2116967119.sapp.pdf]

Supporting Information Appendix

Supporting Information for
Music of Infant-Directed Singing Entrains Infants' Social Visual Behavior

Authors: Miriam D. Lense, Sarah Shultz, Corine Astésano, Warren Jones

Correspondence and requests for materials should be addressed to Miriam.Lense@vanderbilt.edu or Warren.Jones@emory.edu

This PDF file includes:

SI Materials and Methods
SI Supplementary Results
Figures S1 to S5
Table S1
SI References

Supporting Information (SI)

SI MATERIALS AND METHODS **3**

Participants 3
Inclusion / Exclusion Criteria *3*
Initial Study Cohort *3*
Replication Study Cohort *4*
Experimental Design and Stimuli 4
Study Design and Synchronization Terminology *4*
Audiovisual Recordings *5*
Replication Set Stimuli *6*
Reduced Predictability Stimuli *7*
Experimental Procedures and Data Collection 7
Data Processing 8
Identification of Eye Movement Events *8*
Calibration Accuracy *9*
Minimum Valid Data Criterion *9*
Region-of-Interest (ROI) Comparisons *9*
Data Analysis and Statistics 10
Rhythmic Structure and Acoustic Parameters *10*
Motion of Singers *11*
Blinking of Singers *11*
Emotional Expression of Singers *12*
Sample Size *13*
Peristimulus Time Histograms *13*
Phase Analyses *15*
Lissajous Curves *16*

SI SUPPLEMENTARY RESULTS **17**

Fixation Time Comparisons 17
Lissajous Curves: Comparisons of Continuous Time-Varying Signals 18
Caregiver Acoustic Cues 19
Caregiver Visual Cues and Rhythmic Structure 20

SI SUPPLEMENTARY FIGURES/TABLE **24**

SI REFERENCES **30**

SI MATERIALS AND METHODS

This research was based in the Marcus Autism Center, part of Children's Healthcare of Atlanta and the Department of Pediatrics at Emory University School of Medicine. The study protocol was approved by the Institutional Review Board of Emory University School of Medicine (00060097, 00089562). Parents/legal guardians of all infant participants gave informed consent prior to participation.

Infants were shown audiovisual recordings of infant-directed singing. While infants viewed the recordings, their visual scanning was measured with eye-tracking equipment. In relation to the rhythmic structure of the singing, we analyzed the timing of infants' visual fixation to singers' eyes. Analysis of eye movements and coding of fixation data were performed with software written in MATLAB.

Details of participants, experimental procedures and data collection, stimuli, data processing, data analysis and statistics are provided below.

Participants

A total of 145 infants participated in the present studies, 112 in the first set of experiments (across 2 age groups) and 33 in replication (all 6-month-olds), described in greater detail below.

Inclusion / Exclusion Criteria

Infants were enrolled as a representative sampling of typical development. Factors associated with increased risk of atypical development were treated as exclusionary criteria: infants were excluded if they had experienced significant pre- or perinatal complications (i.e., leading to neurological or developmental delays); if there was family history of intellectual or developmental disabilities in first degree relatives; or if there was family history of autism spectrum disorder (ASD) in first, second, or third degree relatives.

Initial Study Cohort

56 two-month-old (mean 2.7 months, SD 0.46, range 1.7-3.4 months, 43% male) and 56 six-month-old (mean 6.2 months, SD 0.38, range 5.5-7.4 months, 57% male) infants participated in the main study. An additional 20 2-month-old participants and 17 6-month-old participants enrolled but had no

usable eye-tracking data collected due to infant fussiness, infant falling asleep during testing, infant failing initial calibration or calibration verification (details below in Experimental Procedures and Data Collection section), or infant failing to meet minimum valid fixation data criterion (see below in Minimum Valid Data Criterion). Usable data were collected from 74% (56 of 76) of enrollees at 2-months, and from 77% (56 of 73) of enrollees at 6-months.

Replication Study Cohort

33 six-month-old (mean 6.2 months, SD 0.36, range 5.0-6.8 months, 52% male) infants participated in and provided usable data in the replication study. Usable data were collected from 89% (33 of 37) of enrollees.

Experimental Design and Stimuli

Study Design and Synchronization Terminology

This study adopts synchronization terminology found in Pikovsky, Rosenblum, & Kurths, 2001 (1). We note that for specificity's sake and also to highlight that—although the current work shares common interests and common conceptual territory with studies of interpersonal synchrony (as reviewed in (2, 3))—our use of the terms synchrony and synchronization is intentionally narrower than some encountered elsewhere in the literature.

Specifically, the current study probes synchronization defined as the *entrainment of an autonomous system by weak external forcing* (see chapter 3 in (1)). Here, the infant is the autonomous system (a dynamical system capable of producing its own independent actions, in this case: the infant's looking behaviors). The singing caregiver constitutes the external force (another independent, autonomous system with capacity to influence rhythms of the first). *Weak* external forcing specifies the strength of coupling between the two. If one system directly controls another, then the two essentially become one, more unified than synchronized (in the present study, weak interaction is confirmed in the infant's ability to look anywhere onscreen, at any time, or not at all). Finally, as noted in the main text, our study design focuses on entrainment of infant behavior rather than on mutual synchronization of infants and caregivers; this is a pragmatic decision to rule out effects of caregiver accommodation.

Use of this narrower definition is not intended as a disconnect from other examples of synchrony (as above); rather, abiding here by a narrower definition allows the behavior of human infants to be studied within a mathematical framework that is common to other studies of elemental entrainment processes (from mechanical and electrochemical coupling (1), to phase-locking of cells in a network (4), to the synchronization of animals' activity (5, 6)).

Audiovisual Recordings

Children watched audiovisual recordings of actresses singing common infant-directed songs (e.g., "Twinkle, Twinkle Little Star", "Old MacDonald"). Nine audiovisual recordings were presented, each with an average duration of 23.6 s (SD = 3.7 s; range 18.2-29.4 s). In total, the 9 audiovisual recordings comprised 227 beats (consistent with song notations). Actresses in each recording were filmed singing directly into the camera (to engage the onlooking child) in front of a background decorated like a child's room, with toys, pictures, and stuffed animals (see **Figure S1A,B**). In each video, the actress's face subtended approximately 15.8° by 12.6° of visual space (horizontal by vertical, with eyes spanning, on average, ~8.0° horizontal by ~6.9° vertical), while bodies subtended ~25.1° horizontal by ~21.7° vertical (presented, as noted above, on a display monitor approximately 24° x 32°). Five different actresses contributed to the stimulus set. Singing videos were interleaved with two other types of stimuli as part of other ongoing experiments not analyzed here (naturalistic scenes of infant-directed speech, as in (7) and scenes of other children at play (8)).

Videos were 640 x 480 pixels in resolution, presented full-screen on a 20-inch computer monitor (refresh rate 60 Hz noninterlaced) at 30 frames per second. Audio (44.1 kHz) was presented in mono-channel. All videos were sound and luminosity equalized, and have been piloted and used successfully in other published studies of infant social engagement (7, 8).

Actresses were non-professional singers, with naturally-occurring variation in tempo, amplitude, and tone, instructed to sing as if they were engaging with an infant: the average inter-beat interval across all songs (strong/weak beat metric structure) was 434 ms (SD=112 ms) (138 beats per minute); the average coefficient of variation was 12.7% (SD=1.9%).

We used audiovisual recordings of infant-directed singing to create an explicit, *uni*directional test of infant entrainment: while coordination of actual infant-caregiver interaction is, of course, bidirectional(9, 10), in our experimental design, infant behavior could have no effect on caregivers (the audiovisual recordings); if the two became synchronized, the effect would necessarily be due to infant entrainment to caregiver cueing (rather than caregiver accommodation). This experimental design is critical for this initial investigation of infant entrainment and lays the groundwork for future studies of mutual entrainment. However, we note that more complex mathematical techniques will need to be employed when investigating dynamic, bidirectionally coupled systems, especially in light of potential confounds of a conscious, accommodating partner (e.g., as demonstrated in more simplified systems in (11–13)); this mutual entrainment of caregiver and infant behaviors that can be either automatic/reflexive or volitional/consciously controlled, are different than those encountered when measuring phase-locking of two signals not under conscious control (such as EEG).

Replication Set Stimuli

For replication, as in the original experiment, children watched audiovisual recordings of actresses singing common infant-directed songs. In replication, children watched 4 recordings with a mean duration of 21.0 s (SD=4.8 s; range 14.9-26.7 s). Fewer recordings were used in replication than in the original experiment due to inclusion of an additional experimental comparison (see below). The average inter-beat interval across replication stimuli was 488 ms (SD=119 ms) (123 beats per minute); the average coefficient of variation was 13.2% (SD=1.0%). As in the original experiment, replication set stimuli were interleaved with two other types of stimuli as part of ongoing experiments not analyzed here (naturalistic scenes of infant-directed speech, as in (7), and scenes of other children at play (8)).

In addition, replication set videos were interleaved with reduced predictability stimuli (see next section below). The experimental design decision to use fewer audiovisual recordings to test for replication (4 rather than 9) was made to allow for additional data collection time to test effects of reduced predictability: because replication of the original entrainment finding is a necessary prerequisite to meaningfully disrupt it, we needed to present both original waveform videos (for replication of the main finding) and reduced predictability stimuli (for the new experiment). Consequently, within the same total

duration of viable testing time for infants, half of the videos presented in replication were original (unmanipulated) audiovisual recordings and half were reduced predictability stimuli.

Reduced Predictability Stimuli

To test whether entrainment in infant eye-looking does or does not depend upon rhythmic predictability of caregiver cueing, we experimentally manipulated original audiovisual recordings to make jittered versions of each recording, resulting in reduced rhythmic predictability. Specifically, we re-sampled the original audiovisual recordings—which had naturally varying but predictable inter-beat intervals—to instead reduce their predictability: in each song, two-thirds of the inter-beat intervals were randomly varied by +/-30% of their original duration, disrupting the original rhythmic structure and reducing beat-to-beat predictability (main text **Figure 4**). The manipulation of inter-beat intervals in audiovisual stimuli was accomplished via granular resynthesis in Ableton Live, simultaneously warping audio and visual signal to ensure fully synchronous audiovisual stimuli. Jittered versions had mean duration of 20.8 s (SD=4.7 s, range 15.4-26.7 s). The average inter-beat interval was 492 ms (SD=118 ms) (122 beats per minute); the average coefficient of variation was 28.4% (SD=5.5%).

It is worth noting that the reduced predictability stimuli, while designed and implemented by means of changes in beat predictability, also provide strong experimental controls for both simple motion effects and for caregiver visual cueing, as the overall motion and visual facial cueing of the caregiver are preserved in the reduced predictability stimuli: i.e., the same range of head and facial motion and all affective cues are preserved and presented, while only their relative temporal predictability is manipulated. Stated differently, the reduced predictability stimuli present the same range in rigid head motion, range in facial feature motion, and range of facial expressions (all in the same spatial locations), as in the original audiovisual recordings, but the predictability in timing of when those events occur is disrupted.

Experimental Procedures and Data Collection

Data collection procedures matched those reported in (7). Infants sat in a reclined bassinet mounted on a table that was raised or lowered to ensure standardized position of infants' eyes relative to

the display monitor (28 inches diagonally, subtending an approximately 24° x 32° portion of the infants' visual field). Lights in the room were dimmed. A parent or primary caregiver accompanied the infant at all times but both the parent and experimenter were out of the infant's view during data collection. Experimenters monitored infants via the eye-tracking camera and a second video camera that displayed a full-face view of the infant. Sessions were stopped before a child completed watching all stimuli if the infant fell asleep or became too fussy to watch the videos. Eye-tracking cameras and an infrared light source were concealed within a teleprompter that displayed the videos while audio was played through speakers mounted at equidistant locations 3" to the left and right of the monitor. Eye-tracking was accomplished by a video-based, dark pupil/corneal reflection technique with hardware and software created by ISCAN, Inc. (Woburn, MA, USA), with data collected at 60 Hz.

Data collection began by presenting soothing but engaging videos to acclimate the child to the testing set-up (e.g., *Baby Mozart*). When the infant was attentive, a 5-point calibration scheme was presented utilizing audiovisual stimuli (spinning and/or flashing lights, cartoon animations, together with accompanying sounds). Calibration stimuli began as large targets (≥10° in horizontal and vertical dimensions) which then shrank (via animation) to their final size of 1° to 1.5° of visual angle. The calibration routine was followed by verification of calibration in which more calibration targets were presented at any of nine on-screen locations. Throughout the remainder of the testing session, calibration targets were shown between experimental videos to measure possible drift in accuracy. After calibration checks, the system was re-calibrated if excessive drift (>3° of visual angle) in calibration accuracy occurred. Please see Data Processing: *Calibration Accuracy* below for measures of calibration accuracy.

Data Processing

Identification of Eye Movement Events

Analysis of eye movements and coding of fixation data were performed with software written in MATLAB (MathWorks). The first phase of analysis was an automated identification of non-fixation data comprising blinks, saccades and any missing data or fixations directed away from the presentation screen. Saccades were identified by eye velocity using a threshold of 30° per sec (14). We tested the velocity threshold with the 60-Hz eye-tracking system described above and, separately, with an eye-

tracking system collecting data at 500Hz (SensoMotoric Instruments GmbH). In both cases saccades were identified with equivalent reliability as compared with both hand coding of the raw eye-position data and with high-speed video of the child's eyes. Blinks were identified as described in (15). Missing data and off-screen fixations (when a participant looked away from the video) were identified either by missing values in gaze vector data or by gaze vectors directed to locations beyond the stimuli presentation monitor.

Calibration Accuracy

Average calibration accuracy for all groups was less than 1° of visual angle. **Figure S1C,D** shows total variance in calibration accuracy, and **Figure S1E,F** shows average calibration accuracy. Calibration accuracy did not differ significantly between age groups (**Figure S1E,F**).

Minimum Valid Data Criterion

For each audiovisual recording, we used a minimum-valid-data criterion of fixation time greater than or equal to 20% of total recording duration, as in (8). We set no thresholds for either minimum number of audiovisual recordings nor minimum number of beat trials sufficient for inclusion of an infant's data in analyses; if usable data were collected, with a given audiovisual recording fixated at a level greater than or equal to the minimum-valid criterion noted, then the infant's data were included. Of 9 possible audiovisual recordings (main experiment), the mean number included for 2-mo-olds was 4.3(1.5) and for 6-mo-olds was 5(2.5) (data given as mean(SD)), t_{110}=1.71, p=0.09). Mean number of beat trials per child at 2 months was 96.9 (38.0) (mean(SD)) and at 6 months was 105.3(55.9) (t_{110}=0.92, p=0.36) (**Figure S1G**).

Region-of-Interest (ROI) Comparisons

Eye movements identified as fixations were coded into four regions of interest (ROIs) that were defined within each frame of all video stimuli as shown in **Figure S1A,B**: eyes (our primary dependent variable in the current study), as well as mouth, body (neck, shoulders and contours around eyes and mouth, such as hair) and objects (surrounding inanimate stimuli). The regions of interest were hand

traced for all frames of each video and stored as binary bitmaps. Automated coding of fixation time to each region of interest then consisted of a numerical comparison of each infant's coordinate fixation location data with the bitmapped regions of interest. From the eye-tracking data, we determined proportion of time spent attending to the video (**Figure S1H**) as well as proportion of time spent fixating on the eye region (**Figure S1I**).

Data Analysis and Statistics

Rhythmic Structure and Acoustic Parameters

We quantified the rhythmic structure of each song by coding vowel durations of all notes in strong metrical positions (i.e., the underlined vowels in '*Twinkle twinkle little star…*'), similar to prior studies of infant-directed song (16, 17). Coding was accomplished by visualization of each speech waveform and spectrogram, as well as by interactive playback (16–19), by two trained and experienced coders, including an expert phonetician, who reviewed and confirmed all codings. Using time stamps from the audio codings of vowel onsets and offsets, we generated frame-by-frame binary time series indicating whether or not corresponding video frames aligned in time with vowels in metrically strong positions (termed 'beats' for brevity). We used vowel durations (rather than only onsets) to quantify rhythmic structure because meaningful social communication requires elapsed time (i.e., the passage of an experiential span of sufficient duration to enable communication transfer).

We also considered other acoustic cues related to rhythmic structure. The rhythmic structure of infant-directed singing necessarily involves multiple inter-related prosodic parameters, including variation in parameters such as pitch and loudness (16). These parameters could play a role in modulating infants' visual attention. We quantified these parameters as follows in order to measure their relationship to infant looking: Acoustic measures of pitch and loudness were calculated as mean fundamental frequency (Hz; proxy for perceived pitch) (20) and root-mean-square amplitude (proxy for perceived loudness)(21), respectively, in time intervals equivalent to the duration of each video frame (i.e., in 33.3 ms bins). For each video, time intervals with fundamental frequency or amplitude values greater than the 90th percentile were used to define time series of "high" frequency or amplitude. As noted in the main text (**Figure 2**), neither high frequency nor high amplitude alone was sufficient in and of itself to drive synchronous infant

eye-looking. To assure that results were not dependent upon threshold selection (90th versus other percentiles), follow-up analyses were conducted with varying thresholds (95th, 92nd, 88th, 85th, 80th percentiles) and yielded consistent results across all comparisons. Note that in infant-directed song, frequency is influenced by the melody of the song; this is in contrast to infant-directed speech, which employs pitch accents for communicative emphasis (22). Amplitude, however, is related to rhythmic structure (16) but also reflects the variable volume (i.e., musical dynamics) used during expressive singing. The goal of the comparative analyses of the effects of different parameters was to test the extent to which discrete occurrences thereof offer evidence for which parameters play a greater (beat) or lesser (any high frequency, high amplitude) role in driving synchronous responding.

Motion of Singers

Motion of the singers was quantified in two ways for two different kinds of motion: motion of the internal features of the face, and rigid motion of the head. To quantify motion of the internal features of the face, we calculated the absolute difference in image intensity (luminance) per video pixel over time. Change in intensity was summed for all pixels in the eyes region-of-interest (ROI) to provide a metric of change within the eye region. We then identified frames with values less than the 10th percentile to define a time series of low motion (i.e., periods relatively free from motion in the eye region). We were interested in periods of low motion as they represent relative stilling. As before, to assure that results were not dependent upon threshold selection, we repeated analyses with additional thresholds (5th, 15th, 20th); results across varying thresholds were consistent with those presented in **Figure 3**.

To quantify rigid motion of the head, we tracked the (x,y) location in video pixel coordinates of the tip of the nose through all frames of all videos. With these data, we could measure up-and-down and side-to-side motion of the head, in relation to the beat and in relation to infant eye-looking. Not surprisingly, up-and-down movements of the head are synchronized with the beat (we found no significant side-to-side head motion versus beat synchrony). Notably, however, increase in infant eye-looking precedes the up-and-down motion of the head, indicating anticipatory looking behavior.

Blinking of Singers

Blinks of the singing actresses were coded manually from each video using frame-by-frame inspection. Timing of blink on- and offsets were coded based on coder's observation of occlusion of the singer's pupils. All blinks were determined by two independent coders, with >99% agreement. Frame-by-frame binary time series were then created to indicate whether or not each video frame aligned in time with a singer's blinking.

Emotional Expression of Singers

In general, when singing to infants, caregivers display positive affect and smiles (23, 24). In our analyses, we were most interested in changing facial expressions reflecting (a) varying levels of caregiver communicative content and (b) varying levels of caregiver engagement, both of which will impact what and how a caregiver conveys information and may also impact infants' attention to a singing caregiver's eyes.

To quantify emotional expressions in the faces of singing caregivers, we used IntraFace software (25). In brief, IntraFace uses feature tracking in videos of faces (via a "Supervised Descent Method" (26)) to track points on a face, and then, based on the positions of those points, quantifies the activity of facial action units (accomplished by an inductive machine learning approach dubbed a "Selective Transfer Machine") to categorize the resultant patterns into generic facial expressions. The result is a quantification of facial action unit activity and a probability rating of emotional expression for every frame of video. [Note: When these analyses were conducted, Intraface Software was freely available for research use; it was subsequently acquired by Facebook and is no longer publicly available. OpenFace is a comparable package that can be found at https://cmusatyalab.github.io/openface/ .]

Analyses focused on variation in two facial expressions: neutral, which involves relaxed eyes/brows (the absence of facial action unit activity; IntraFace's "neutral" classification), and "mock-surprise"/wide-eyed engagement, which involves raising of the upper eyelids and brows (action units 1, 2, 5; IntraFace's "surprised" classification). The "surprised" classification from IntraFace is consistent with the canonical expression of surprise in adults but also with an expression called "mock-surprise" or the "wow" expression that commonly occurs in infant-directed communication (27, 28). This infant-directed mock-surprise involves raised eye action units (wide open eyes, raised eyebrows) and open mouth, and

is rated as expressing surprise, excitement, and interest by naïve raters (28). It is worth noting that mock-surprise, despite being extremely common in caregiver-infant interaction (29, 30), and immediately known to most parents and caregivers, is rarely mentioned in the adult facial expression literature (31): rather, mock-surprise exists specifically within the developmental context of infant-caregiver interaction (one of multiple such acts that emerge and exist specifically within the context of dyadic interaction with infants (30)).

To test whether IntraFace facial expression classifications were consistent with human observer perceptions, 10 naïve adults rated the emotional expressions of video frames pseudo-randomly selected from all videos (selected pseudo-randomly to ensure a variety of expressions; 36 ratings for each of 10 coders). Frames classified as "surprised" by IntraFace were consistently rated as higher in surprise, wide-eyed engagement, excitement, and interest than non-surprised frames ($t(349)$'s$\geq$6.44, p's<0.001), confirming the reliability of the software's surprise classification.

For each video, Intraface ratings were used to define frame-by-frame time series indicating presence or absence of the expression of interest (either neutral expression or wide-eyed engagement).

Sample Size

For determining sample size in the present study, power calculations were based on data from the existing literature on infant eye-looking(7) (including expected frequency and duration of eye-looking) and on the expected observable effect size modeled as the strength of observable association between caregiver action and hypothesized infant eye-looking response (correlation between inter-onset intervals of action and response). Analyses indicated that samples of 50 or greater would provide 80% power to detect effects with magnitude equal to approximately 0.34 (α = 0.05). Measurement estimates of our achieved power (1-β error probability) for 2-mo-old entrained eye-looking was 0.86; in 6-mo-olds, achieved power for entrained eye-looking was 0.99. Given the large effect size observed in 6-mo-olds in the original experiment, in our replication study (**Figure S5** and **Figure 4**), we relaxed the sample size required to N = 30 or greater.

Peristimulus Time Histograms

Peristimulus time histograms (PSTHs) were used to determine timing of fixations to the eyes relative to timing of the stimulus events of interest (i.e., to beats (vowels aligned with strong metrical positions), acoustic parameters, facial expressions) following the methods detailed in (15). Repeated here in brief, PSTHs were constructed by aligning individual binary time-series data for each infant's fixations to the eye ROI (0 = not fixating on eye ROI; 1 = fixation on eye ROI) with the binary time series for the relevant stimulus event (0 = not stimulus event; 1 = stimulus event). We counted fixations to the eye region in 33.3-ms bins in a window from -433.3 to +433.3 ms around the stimulus event. Bin counts were totaled across all events and for each infant and then averaged for group means at 2 and 6 months of age.

We used permutation testing to examine if change in eye looking synchronized to the stimulus event differed from change expected by chance. Binary times series for each infant were permuted by circularly shifting the time series by a random number for 1000 iterations. This approach preserves overall frequency and duration of fixations to the eye ROI for each infant but makes the fixations random with respect to the time course of the stimulus events of interest and to other infants' fixations. The mean of the permuted data represents chance-level fixation data relative to the stimulus event. We compared actual fixation data against the 95th percentile of the permuted data to test for significant increases (one-sided test, α=0.05) in eye-looking time-locked to the stimulus event of interest in the singing (e.g., beats, high frequency, high amplitude, emotional expression, etc.). This same approach was taken to assess time-locking of acoustic (high frequency, high amplitude) and visual (low motion, blinks, emotional expression) prosodic markers of the infant-directed singing at the beats, using time-series of the relevant prosodic marker (0 = no prosodic marker; 1 = prosodic marker) relative to time-series of the rhythmic structure (0 = no beat; 1 = beat).

To examine whether PSTH magnitude and shape were significantly greater for 6-month-old versus 2-month-old infants, we again used permutation testing. In 10,000 random re-samplings, we repeatedly created two groups of independent infants, randomly selected across all 6-month-old and 2-month-olds, and then computed their between-group difference in PSTHs. The mean difference across all 10,000 permuted samples represents chance-level difference at each time point. We then compared the actual observed 6- versus 2-month between-group PSTH difference against the 95th percentile of PSTH

differences expected by chance alone (one-sided test, alpha=0.05). That comparison enabled us to test whether time-locked eye-looking at 6 months was significantly greater than at 2 months.

Phase Analyses

To estimate each infant's phase of response, ϕ, at the beat, each infant's PSTH data were first fitted with each of 3 models (fitting via nonlinear least squares method). The data were fitted with a simple linear function (1st degree polynomial, $y = ax + b$), with a cosine function ($y = \cos(ax + b)$), and with a cosine function with additive linear trend ($y = \cos(ax + b) + cx + d$) (with, in each case, y denoting an individual's level of response, x denoting time, and a, b, c, and d denoting coefficients of the respective fitted function). Among the three fits, the best-fitting function was selected by goodness-of-fit statistic (R^2 coefficient of determination).

When comparing results from each of the three fits, to be conservative in our analyses, we interpreted cases in which the simple linear fit (1st degree polynomial) produced the highest goodness-of-fit statistic as indicating that there was no reliable evidence of a phasic response for that infant in a given condition. Stated simply: if the data were best fit by a straight line, there was no reliable statistical evidence for phasic response. With no reliable evidence of a phasic response, that infant's data were excluded from further phase analyses for that condition (number of exclusions reported in **Supplementary Table 1**). Note that exclusion from individual phase estimation occurred in only 1.75 infants per condition (mean(SD) = 1.75(1.4)), and only affected phase estimation analyses and plots for that individual infant for that condition; no other conditions or analyses were affected, and group metrics and group PSTHs in all conditions include data from all infants. As seen in **Supplementary Table 1**, goodness of fit statistics for phase analyses were very high across all conditions (R^2 >0.81 in all conditions), with the vast majority of infants' data fitted successfully with a cosine function and only a small number (no more than 4 infants in any condition) with no statistical evidence for phasic response.

In cases when individual children's data were better fit with a cosine function (when there was evidence of individual phasic response for a given child for a given condition), then that infant's phase of response at the beat, ϕ, was calculated as the local maximum closest in time to 0, obtained by solving for zero on the first derivative of the fitted function: $\phi = {-b}/{a}$ (for $y = \cos(ax + b)$ as the best fitting

function) or $\phi = (\arcsin(^{c}/_{a}) - b)/a$ (for $y = \cos(ax + b) + cx + d$ as the best fitting function). These individual infant ϕ estimates are plotted as inset graphs in Figures 2-4, S3-S5.

To analyze distributions in ϕ estimates, we used circular statistics. We assessed synchronization of eye-looking response with the beat using the V-test, testing for non-uniformity of ϕ distributions around 0 (32, 33). To compare tightness of phase-locking between the two- and six-month groups (i.e., consistency of response among individuals), we used the Wallraff test of angular dispersion (32, 34).

Lissajous Curves

As a complementary method for observing synchronization between the beat of infant-directed singing and the looking behavior of infants, we constructed Lissajous curves comparing the changing phase of the beat with the varying probability of infant-looking behavior. Lissajous curves provide a direct record of how two time-varying signals vary in relation to one another, and Lissajous curves can be used to visualize synchronization between two continuous signals, to quantify phase shift from one signal to another, and to identify higher order synchronization (e.g., 2:1, 3:1,… n:m frequency coupling) (**Figure 1L**).

In these analyses, the phase of the beat was estimated as a continuously varying cosine function as plotted in **Figure 1D**. As noted in the main text and described above in "Rhythmic Structure and Acoustic Parameters" section, beats were coded and quantified as the vowel durations of all metrically strong syllables within each song. With manually-labeled beats in all songs, the corresponding cosine function was calculated to reach a local maximum at the midpoint of each labeled beat and to reach a local minimum value at the midpoint of each between-beat interval.

To quantify probability of infant-looking behavior, the probability of a given behavior was defined as the number of infants performing that behavior (numerator) divided by the total number of infants who could have been performing that behavior (denominator): for example, probability of infant eye-looking equaled the number of infants looking at a singer's eyes divided by the total number of infants who could have been looking at a singer's eyes. That quantification was repeated at each moment (sample) in the time series to quantify time-varying probability of infant behavior for the entire time series in all audiovisual recordings. To smooth the data and normalize for global variance in number of infant viewers,

we computed two filtered versions of each behavioral time series: one filtered with a moving-average square window of 12 samples (local window, corresponding to 400 ms of the time series, for low pass filtering), and a second with a square window of 60 samples (global window, corresponding to 2 sec of the time series, for high pass filtering). We then subtracted the signal filtered at the local window from the signal filtered at the global window, normalizing for global variance in intensity while preserving local signal change(35). Finally, the local and globally filtered signal was standardized to have the same mean and variance as the original (unfiltered) signal.

With that measure of time-varying probability of infant-looking behavior, together with the cosine function specifying phase of the beat across all songs, we were left with two continuous time-varying signals that could be directly compared by plotting as Lissajous curves. Paradigmatic examples of non-synchronous and synchronous relationships between 2 signals are plotted in **Figure 1L**.

SI SUPPLEMENTARY RESULTS

Fixation Time Comparisons

As noted above in the Data Acquisition and Processing section, although our primary dependent variable was fixation on caregivers' eyes, infant eye movements identified as fixations were coded into four regions of interest defined within each frame of all video stimuli: eyes, mouth, body (neck, shoulders and contours around eyes and mouth, such as hair) and objects (surrounding inanimate stimuli) (**Figure S1A,B**).

Infants at both ages had similar proportions of overall time spent fixating (mean(SD) at 2-mos: 59.4%(16.4); 6-mos: 63.7%(13.5); t_{110}=1.50, p=0.14) (**Figure S1H**), as well as proportion of time spent fixating on the eyes (2-mos: 31.3%(22.6); 6-mos: 31.6%(19.2); t_{110}=0.06, p=0.95) (**Figure S1I**). There were no significant differences in proportion of time spent in eye-looking between the two age groups. That absence of significant differences contrasts somewhat with results from our earlier work (7), in which we observed an increase in eye-looking between 2- and 6-mo-old males followed longitudinally. Notably, however, the current comparisons differ in 3 ways from those prior results. First, the results here are for infant-directed singing, not speech. Second, the results here are for independent-sample, between-subjects, cross-sectional comparison of means rather than a longitudinal within-subjects comparison of developmental change. And third, the current sample includes both males and females rather than males

alone in (7), and when followed longitudinally, females increase their eye-looking more rapidly than males, from 2 until ~4 months, before then decreasing slightly from ~4 to 6 months; in contrast, males increase looking more slowly from 2 until 6 months, as in (7).

Lissajous Curves: Comparisons of Continuous Time-Varying Signals

Complementary analyses of synchronization compared continuously-varying measures of the changing phase of the beat with continuously-varying probability of infant-looking behavior by constructing Lissajous curves (**Figure S2**).

Beginning with the 6-month data, as shown in **Figure S2C**, the resulting Lissajous curves, like the main text PSTH analyses, show evidence of synchronization between infant eye-looking and the beat of infant-directed singing: probability of 6-month-old infant eye-looking increases in synchrony with the beat, with 1:1 synchrony and phase shift of ~π/5.5 (phase shift = 0.5669; eye-looking probability is maximally increased slightly after the beat, as shown in the time-/direction-annotated traces at right of panel **Figure S2C**). Probability of mouth-looking (**Figure S2E**) also shows 1:1 synchrony with similar phase shift (~π/5.5, 0.5729), but is synchronous in anti-phase, maximally reduced after the beat. Variation in probability of 6-month-old body-looking (**Figure S2G**) shows no evidence of synchrony: probability of body-looking does not vary systematically in relation to the beat. Saccades, by contrast, are synchronized at 2 saccade periods per 1 beat period, with maximum increase just prior to the beat (**Figure S2I**), indicating an increase in saccades occurring in anticipation of the beat (phase shift ahead of the beat by ~ -π/10.3, phase shift = -0.3055)).

Lissajous curves for 2-month-olds show similar but developmentally attenuated synchronization. Similar to 6-month-olds, probability of 2-month-old infant eye-looking increases in synchrony with the beat (**Figure S2B**), with 1:1 synchrony and phase shift of ~π/4.8 (phase shift = 0.6500). Mouth-looking also shows 1:1 synchrony in anti-phase (**Figure S2D**), maximally reduced prior to the beat, but is more phase-shifted in 2- than 6-month-olds: ~π/2.97 vs ~π/5.5, respectively. As with 6-month-olds, variation in probability of 2-month-old body-looking does not vary significantly with the beat (**Figure S2F**). Finally, the Lissajous curve for 2-month-old saccade probability appears to show early developmental transition

towards 2 saccades per 1 beat period, but only weakly so, approaching ~2:1 coupling and phase-shifted by ~ $\pi/2.82$ (**Figure S2H**).

All Lissajous curves plotted in **Figure S2B-I** show average probability across all beat trials, with variance in beat-by-beat response indicated by gray shading, which shows +/-1 standard error of the mean.

Caregiver Acoustic Cues

Infant-directed communication is well-known for its properties of heightened fundamental frequency, greater pitch contours and variability, longer pauses, slower tempo, and increased repetition(16, 22, 36). Prior research indicates that these acoustic features of infant-directed communication capture and maintain infants' attention (e.g., high fundamental frequency, (22, 37)). However, when we specifically examine moment-by-moment drivers of infant visual attention to the eyes of an engaging caregiver, infant eye-looking was time-locked to the rhythmic structure (beats) but was not significantly time-locked to moments of high frequency or high amplitude (main text **Figure 2**). These results are not in contradiction with the general importance of pitch and loudness in infant-directed communication; rather, they offer evidence that during infant-directed singing, rhythm organizes those and other features. The lack of time-locked looking to high amplitude or frequency events may be due to the context of infant-directed song. During song, the singer's use of volume for expressiveness (i.e., musical dynamics) impacts amplitude levels while melodic contours dictate frequency patterns. Individual notes also exhibit greater pitch stability in infant-directed singing compared to infant-directed speech (38, 39). Thus the use of specific acoustic parameters in song contrasts with the role of pitch accents in contributing to rhythmic structure during infant-directed speech, during which high pitch and pitch variability capture infants' attention (40, 41). The global prosodic frequency and amplitude contours of song may have rendered these specific acoustic cues less relevant for dynamically modulating infants' eye gaze on a moment-by-moment basis. Even while cues such as high frequency are important for attracting infants' overall attention, including during infant-directed singing (22, 37), the precise timing of infants' attentional allocation to a singing caregiver's eyes is more strongly influenced by rhythm than by other acoustic cues. Previous studies of non-infant-directed singing (e.g., professional or layperson

singing performances directed toward other adults) indicate that when acoustic cues are constrained due to the musical/singing context (e.g., by melodic contour), they may be less informative for socio-communicative judgments: when pitch level is controlled, naïve observers are less accurate at identifying specific emotions in audio-only versions of singing versus visual-only or audiovisual versions (42) and the identified emotions are also perceived less intensely in audio-only formats (43). Additionally, as rhythm and other acoustic elements (e.g., pitch) are intertwined for the listener during song perception (44–47), the temporal organization provided by the beat-based rhythmic structure constrains pitch and melody perception: rhythmically shifting a song so that specific pitches are or are not aligned with the beats changes the perceived tonality and reduces recognition of pitches (even if the pitches themselves are unchanged (44, 45)). This is consistent with rhythm as a temporal organizer of listeners' experiences: rhythm plays an important role in structuring and scaffolding experience when engaged with song.

All stimuli in the current study were common children's songs performed in an infant-directed manner (i.e., higher fundamental frequency) to be developmentally appropriate for our sample and research questions. Future studies could use specifically constructed melodies performed at multiple different pitch levels to further examine effects of high frequency when controlling for the rhythmic structure in which frequency is embedded.

Caregiver Visual Cues and Rhythmic Structure

Rhythm is a salient cue to infants because it is expressed amodally (48, 49). As demonstrated in current data in main text **Figure 3**, caregivers unconsciously structure their own visual cueing in time to the rhythm of their singing, redundantly and repeatedly highlighting infant-relevant communicative cues. Because caregivers use these cues to engage their infants socially, especially during infant-directed singing, a key question is whether these cues drive infant behavioral response independently (i.e., are sufficient on their own), or if infant response relies on or benefits from the redundant, repeated structure provided by rhythm and entrainment (in order to ultimately, most effectively engage infant behavior). A related question is how this confluence of cueing affects multimodal social information transfer developmentally, to support children's social adaptive learning over time.

A way to probe each of these questions is to test the extent to which infant responses vary as a function of different components of caregiver cueing and the extent to which responses vary developmentally. We hypothesized that by imposing a structure to the interaction, rhythm may support other cueing signals by enabling predictable and repeated presentation of multimodal social information, and that these effects should strengthen over developmental time.

To test, we compared entrainment of infant eye-looking during the following conditions: during all beats; during beats *without* co-occurring wide-eyed, positive affect, and during beats *with* co-occurring wide-eyed, positive affect (**Figure S3**). In both 2- and 6-month-old infants, entrainment is evident during all beats (**Figure S3A,D**, results repeated from **Figure 2A,B**). However, a developmental progression is apparent when we separate instances when beats either co-occur or not with a caregiver's presentation of wide-eyed, positive affect: at 2 months, entrainment is driven by the beats, with no effect for co-occurring presentation of wide-eyed, positive affect (**Figure S3B,C**); at 6 months, however, the timing of infant looking is aligned with the beat but also potentiated by a caregiver's presentation of co-occurring wide-eyed, positive affect (**Figure S3E,F**). With development, precise time-alignment of eye-looking behavior is supported by the rhythmic structure of multiple redundant cues.

While these findings in 2-month infants may seem surprising, closer inspection of individual phase responses provides some indication of why this may be. As depicted in **Figure S3A**, while 2-month-olds entrain to the beat, there is also variability in infants' precise individual response timing, with some 2-month-olds aligning just prior to, and others just after, the beat. This variability is consistent with the increased variability in latencies to saccade onset observed in control comparisons between 2- and 6-month-olds (**Figure S1K**), and would be consistent with less mature motor control in 2-month-olds. By comparison, individual 6-month-olds are less variable in their individual time alignment with the beat (**Figure S3D**). We can then compare the slightly increased variability in 2-month-old response with the time-alignment of caregivers' wide-eyed positive affect (also in relation to the beat; i.e., comparing time-alignment of infant response to the beat versus time-alignment of caregiver behavior to the beat). Time alignment of caregiver wide-eyed positive affect with the beat (main text **Figure 3A**) is much more precise, tightly aligning with or just prior to the beat. We think it likely that the slightly increased individual

variability in 2-month-old time-aligned eye-looking, coupled with the precise time-alignment of caregivers' own synchronized expressions, leads to the pattern of observed results.

This developmental progression, aided by infants' maturing oculomotor function, suggests that the rhythm of infant-directed communication provides a scaffolding mechanism for increasing the effectiveness of social information transfer, supporting infants' developing sensitivity to meaningful social signals by presenting those signals repeatedly and predictably. To test for further evidence of rhythm as the primary driver of infants' entrained eye-looking to caregivers' social-affective cueing, we also examined whether infants time-align their eye-looking to *any* moments of caregivers' wide-eyed positive affect (i.e., regardless of whether such expressiveness occurs on or off the beat). While caregivers increase wide-eyed positive affect in time with the beat, this visual cue also occurs at other times throughout their singing. At neither two nor six-months of age do infants significantly time-align their eye-looking to this social-communicative cue when it occurs irrespective of the rhythmic structure (**Figure S4**). (We highlight that these results focus on time-aligned change in levels of infant eye-looking in relation to a given caregiver cue, rather than infants' overall levels of eye-looking. Therefore, these results do not imply that infants don't look at caregivers' wide-eyed positive affect (they do); rather, these combined results demonstrate that the precise timing of infant-looking is time-aligned to the rhythmic structure more than to caregivers' affect presentation alone). Taken together, the analyses of caregiver visual cueing, both overall and in relation to rhythmic structure, indicates that although *what* a caregiver expresses in unimodal cueing is important, *when* and *how* that cueing occurs are more critical for the infant's response and receipt of information. Rhythm—to specify the "when" of predictable repetition—and rhythmic entrainment—to specify the "how" of complementary redundancy—seem ideally suited to the task of supporting successful social information transfer between caregiver and child.

Beyond infant-directed singing, visual communicative cues including eye contact, head movements, and facial expressiveness are important in other performative musical contexts (42, 50–53). Visual cues involving the eyebrows, lips, jaws, and head positioning covary with aspects of the musical structure (e.g., facial movements provide cues to pitch intervals, phrase closure, and amplitude of the vocal signal (54–56)) while also conveying associated emotions (e.g., eyebrow raises, forward head movements, and upward lip corner movements are associated with positive emotions during singing as

they are in speech, highlighting the cross-modal expression of cues during musical performances (42, 57)). Indeed, visual displays are particularly salient for expressing emotions during singing (more so than isolated acoustic counterparts) (42, 43). Observers perceive greater communication and expressiveness from performers who use direct gaze, and this increases the observers' liking and emotional judgments of the performance (50). Some professional musicians are particularly well-known for their expressive visual cues during performances (e.g., (53)). It is possible that in musical performances more generally, the expressive visual cues will be time-aligned to the rhythmic structure as demonstrated in the infant-directed singing. At the same time, the use of such expressive cues and their timing will depend on multiple aspects related to the song requirements, performer attributes, and audience (e.g., (58, 59)). Regardless, it is remarkable that when engaging with infants, who have limited communicative skills and require external support to modulate their attention and arousal, caregivers adopt the highly expressive and engaging visual cues used in performative contexts.

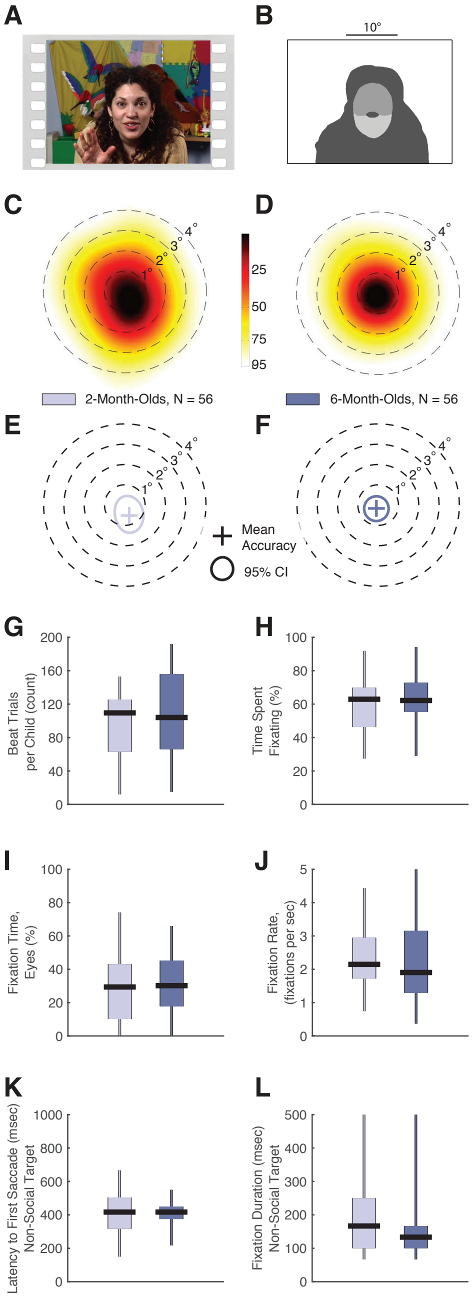

Supplementary Figure S1 | Between-group controls for task completion and calibration accuracy. To test for group-wise differences in quality of data and task completion, we compared calibration accuracy, number of beat trials per child, fixation time, and fixation rate. (**A**) Example still image from infant-directed singing video stimuli. (**B**) Regions of interest, shaded to indicate eyes, mouth, body, and object regions, for the still image in (A) (as coded for all frames of all infant-directed singing videos). (**C**, **D**) Total variance in calibration accuracy for 2-month-olds (C) and 6-month-olds (D). Plots show kernel density estimates of the distribution of measured fixation locations relative to calibration accuracy verification targets. (**E**, **F**) Average calibration accuracy for 2-month-olds (E) and 6-month-olds (F). Crosses mark the location of mean calibration accuracy, while annuli mark 95% confidence intervals (CI). (**G**) Number of beat trials per child with valid data. (**H**) Percentage of total time spent fixating. (**I**) Percentage of time spent fixating on eyes. (**J**) Fixation rate. (**K**) Latency to first saccade when presented with a non-social target. (**L**) Fixation duration following first saccade when presented with a non-social target. In (K-L), we measured latency to first saccade after stimulus onset and the duration of first fixation as additional measures of oculomotor control. While 2- and 6-month-olds do not differ in mean or median latency to first saccade, they do differ in variance in saccade latency, with 2-month-olds being more variable than 6-month-olds ($F_{1,107}$ = 15.9, p < 0.0001; Levene's test for equality of variance). In (G-L), boxplots span full range of data collected, with horizontal black lines marking medians, boxes spanning the 25th to 75th percentiles, and vertical lines extending from minimum to maximum values.

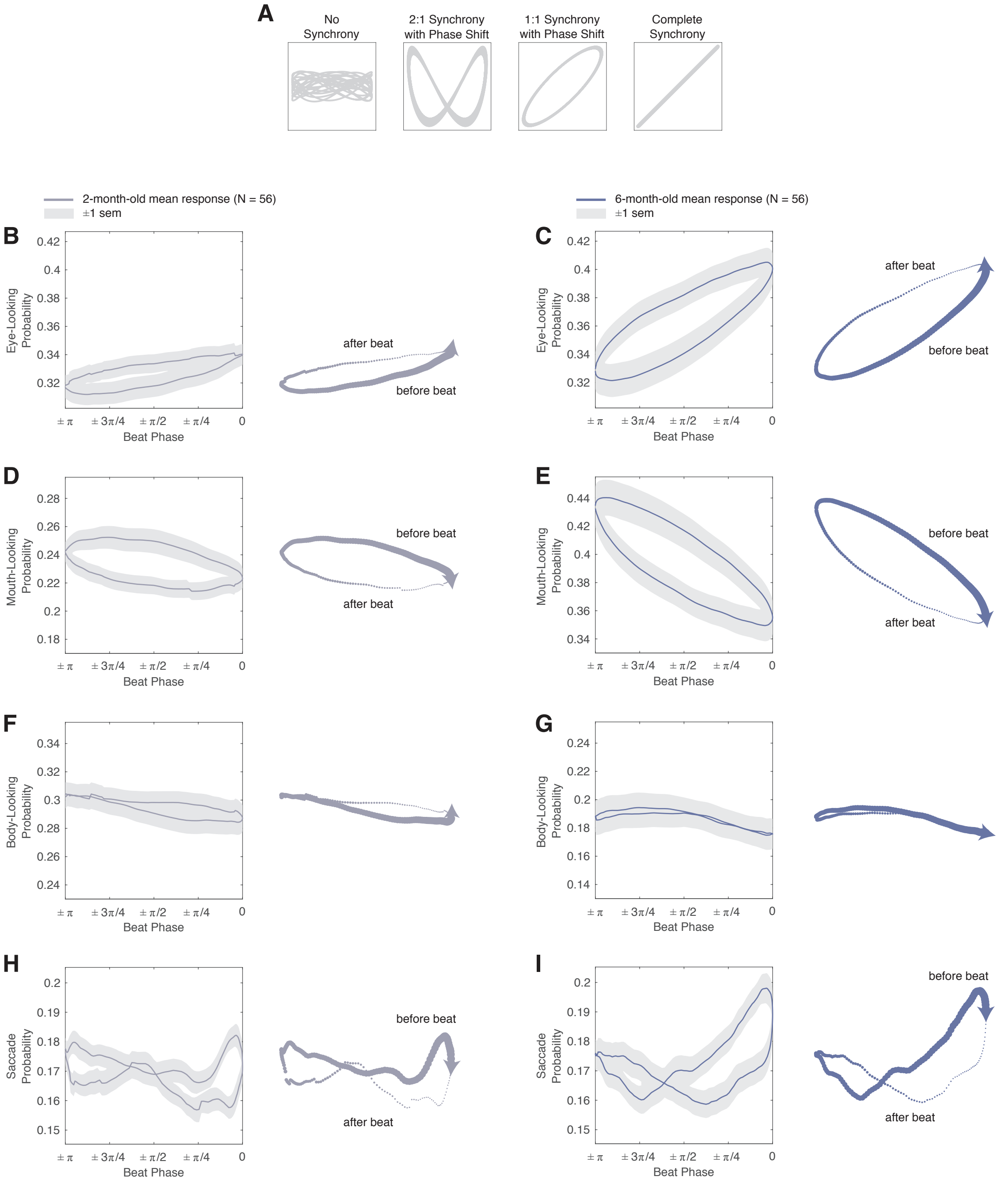

Supplementary Figure S2 │ Lissajous curves show synchronization of infant-looking and beat phase, with increased eye-looking sustained after the beat and increased saccades prior to the beat. (**A**) Exemplar Lissajous curves demonstrating results for varying cases of synchrony between 2 time-varying signals: from no synchrony; to higher order synchrony with phase shift (here, 2 periods of output signal correspond to 1 period of modulating signal); to 1:1 phase synchrony (synchronized with 1:1 periods but with phase shift in timing); and complete synchrony (1:1 synchrony with 0 phase shift). (**B**, **C**) Lissajous curve for probability of infant eye-looking versus beat phase for (B) 2-month-old and (C) 6-month-old infants. Traces at right of each panel show direction of Lissajous curve travel over time. (**D**, **E**) Probability of infant mouth-looking versus beat phase for (D) 2-month-old and (E) 6-month-old infants. (**F**, **G**) Probability of infant body-looking versus beat phase for (F) 2-month-old and (G) 6-month-old infants. (**H**, **I**) Probability of infant saccades versus beat phase for (H) 2-month-old and (I) 6-month-old infants. In Lissajous curves in parts (B-I), mean looking probability is plotted in blue while gray areas denote ±1 standard error of the mean (sem). In all traces, the arrowhead denotes mean response level at the beat (beat phase = 0), with trace thickness denoting direction of travel (thickening as time moves forward, resetting immediately after the beat). Y-axis ranges in parts (B) and (C), and in parts (H) and (I) are the same, whereas Y-axis spans are the same in parts (D) and (E), and (F) and (G), but their ranges differ. Mean probabilities of mouth and body-looking differ between groups; spans are matched for between-group comparison but ranges necessarily differ. Note that a Lissajous curve when no synchrony is present fills the plot area, and the average response probability is unchanged relative to the beat (a horizontal line, with no significant output signal change relative to beat phase, as observed for body-looking in (F) and (G)). Probability of eye- and mouth-looking in 6-month-old infants both show 1:1 synchrony with ~π/5.5 phase shift; however, mouth-looking is synchronous in anti-phase (maximally reduced after the beat). Saccades in 6-month-olds are synchronized at 2 saccade periods per 1 beat period, with maximum increase prior to (in anticipation of) the beat. When comparing synchronization of eye- and mouth-looking at 2-months (left columns) and 6-months (right columns), note greater magnitude of change in probability for 6-month-olds. Similarly, 6-month-olds exhibit greater increase in probability of saccades before the beat versus 2-month-olds.

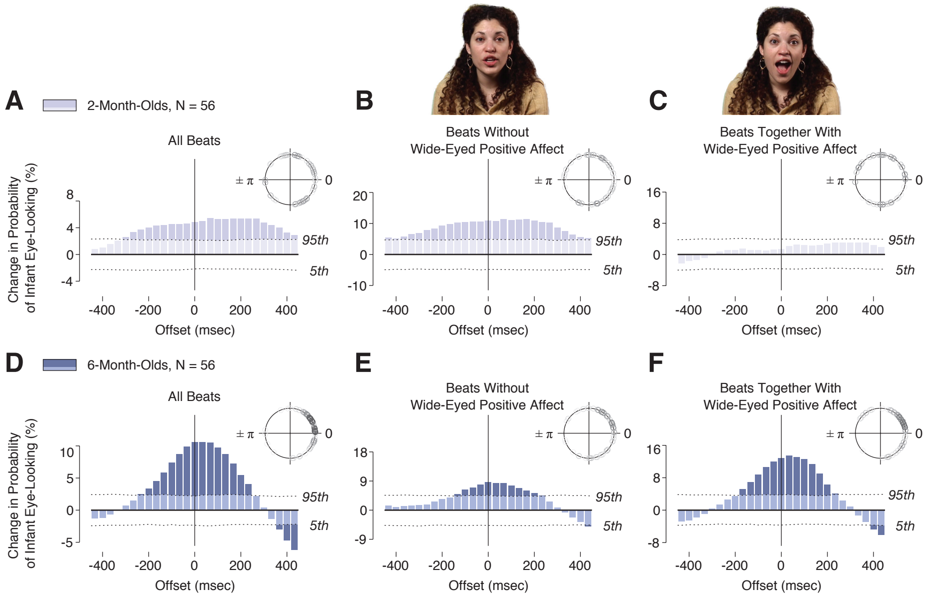

Supplementary Figure S3 │ Developmentally, the rhythm of infant-directed singing increases time-locked looking to relevant social information. Caregiver singing stimuli were intended to create positive engagement with on-looking infants. At 2 months of age, infant eye-looking (**A**) increases at the beat (data repeated from Figure 2a) and (**B**) is driven more strongly by the beat alone than by (**C**) beats co-occurring with wide-eyed positive affect. By 6 months of age, however, infant eye-looking (**D**) is not only significantly increased at the beat (data repeated from Figure 2b), but (**E**) shows tight time-locking to the beat alone and (**F**) is strongly potentiated by beats co-occurring with wide-eyed positive affect. The developmental progression suggests that infant-looking becomes increasingly sensitive to added layers of social information that are supported by the rhythm of infant-directed communication. Dotted lines show 5th and 95th confidence intervals for change in eye-looking expected by chance (1-sided); plots are scaled to align by probability of observed results. Inset plots in the upper right of each panel show phase distributions of eye-looking for individual infants. Images above panels (B) and (C) are representative video stills for each analysis: moments when beats co-occur with wide-eyed positive affect, in (C) and (F), or when co-occurring predominantly with neutral facial affect, in (B) and (E).

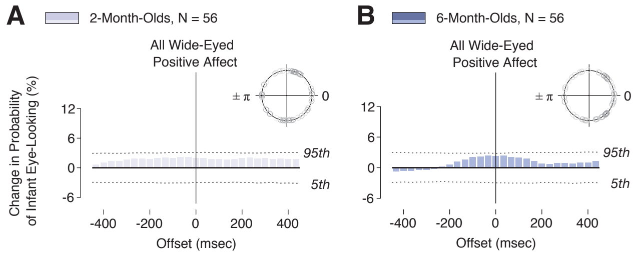

Supplementary Figure S4 │ Infant eye-looking is not time-aligned to all moments of wide-eyed positive affect. During infant-directed singing, singers use positive, engaging facial expressions. However, in both 2-month-old (**A**) and 6-month-old (**B**) infants, eye-looking is not time-locked to all moments of such wide-eyed positive affect from the singer. Note, these findings do not imply that infants do not look at wide-eyed positive affect; rather, they indicate that the precise timing of infant-looking is not time-aligned to the caregiver affective facial expressions alone.

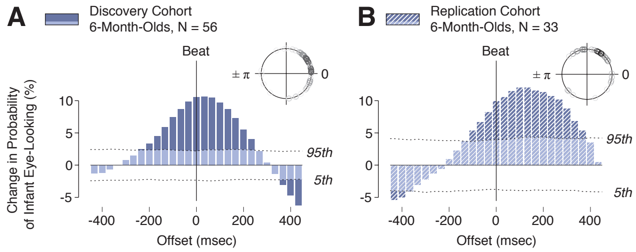

Supplementary Figure S5 │ Replication of Increased Eye-Looking, Synchronized to the Rhythm of Infant-Directed Singing, in an Independent Cohort of 6-Month-Olds. As in the discovery cohort (**A**) (results repeated from main text Figure 2b), in an independent cohort of 6-month-olds (**B**), we again observe significant change in infants' eye-looking, time-locked to the beat of infant-directed singing: infants increase their looking to singers' eyes, time-aligned to the beat and peaking approximately 100 msec after the beat. Dotted lines in both panels show 5th and 95th confidence intervals for change in eye-looking expected by chance (1-sided). Note that the difference in sample size in the replication cohort (N = 33 versus discovery cohort N = 56) is reflected in the confidence interval scaling (the absolute scale is the same while the size of the confidence interval is larger for the smaller replication sample). Inset plots in the upper right of each panel show phase distributions of eye-looking for individual infants.

Supplementary Table 1. Goodness of Fit for Phase Analyses

	Beat	*High Frequency*	*High Amplitude*	*Beats w/o Wide-Eyed Positive Affect*	*Beats with Wide-Eyed Positive Affect*	*Replication*	*Reduced Predictability*
successfully fit[1]*, 2 months*	100.0% (56/56)	96.4% (54/56)	92.9% (52/56)	100.0% (56/56)	100.0% (56/56)	N/A	N/A
successfully fit[1]*, 6 months*	98.2% (55/56)	96.4% (54/56)	98.2% (55/56)	96.4% (54/56)	96.4% (54/56)	87.9% (29/33)	90.9% (30/33)
median[2] R^2*, 2 months*	0.92	0.88	0.82	0.81	0.85	N/A	N/A
median[2] R^2*, 6 months*	0.96	0.91	0.86	0.89	0.94	0.96	0.94

[1] = Percentage of children (and count) whose data were better fit with a cosine than simple linear function.
[2] = Median individual goodness-of-fit statistic, R^2, across all children whose data were successfully fitted.

SI REFERENCES

1. A. Pikovsky, M. Rosenblum, J. Kurths, *Synchronization: A Universal Concept in Nonlinear Sciences* (Cambridge University Press, 2001).
2. S. Hoehl, M. Fairhurst, A. Schirmer, Interactional synchrony: signals, mechanisms and benefits. *Soc. Cogn. Affect. Neurosci.* **16**, 5–18 (2021).
3. R. Feldman, Parent-infant synchrony and the construction of shared timing; physiological precursors, developmental outcomes, and risk conditions. *J. Child Psychol. Psychiatry Allied Discip.* **48**, 329–354 (2007).
4. A. Sirota, *et al.*, Entrainment of Neocortical Neurons and Gamma Oscillations by the Hippocampal Theta Rhythm. *Neuron* **60**, 683–697 (2008).
5. T. Fuchikawa, A. Eban-Rothschild, M. Nagari, Y. Shemesh, G. Bloch, Potent social synchronization can override photic entrainment of circadian rhythms. *Nat. Commun.* **7**, 11662 (2016).
6. S. H. Strogatz, D. M. Abrams, A. McRobie, B. Eckhardt, E. Ott, Crowd synchrony on the Millennium Bridge. *Nature* **438**, 43–44 (2005).
7. W. Jones, A. Klin, Attention to eyes is present but in decline in 2-6-month-old infants later diagnosed with autism. *Nature* **504**, 427–31 (2013).
8. J. N. Constantino, *et al.*, Infant viewing of social scenes is under genetic control and is atypical in autism. *Nature* **547**, 340–344 (2017).
9. H.-C. Hsu, A. Fogel, Stability and transitions in mother-infant face-to-face communication during the first 6 months: a microhistorical approach. *Dev. Psychol.* **39**, 1061–1082 (2003).
10. B. Beebe, *et al.*, A systems view of mother–infant face-to-face communication. *Dev. Psychol.* **52**, 556–571 (2016).
11. B. Kralemann, *et al.*, In vivo cardiac phase response curve elucidates human respiratory heart rate variability. *Nat. Commun.* **4** (2013).
12. M. G. Rosenblum, A. S. Pikovsky, Detecting direction of coupling in interacting oscillators. *Phys. Rev. E. Stat. Nonlin. Soft Matter Phys.* **64**, 4 (2001).
13. B. Kralemann, L. Cimponeriu, M. Rosenblum, A. Pikovsky, R. Mrowka, Phase dynamics of coupled oscillators reconstructed from data. *Phys. Rev. E. Stat. Nonlin. Soft Matter Phys.* **77** (2008).
14. R. J. Leigh, D. S. Zee, *The Neurology of Eye Movements*, 4th Ed. (Oxford University Press, USA, 2006).
15. S. Shultz, A. Klin, W. Jones, Inhibition of eye blinking reveals subjective perceptions of stimulus salience. *Proc. Natl. Acad. Sci. U. S. A.* **108**, 21270–21275 (2011).
16. L. J. Trainor, E. D. Clark, A. Huntley, B. A. Adams, The acoustic basis of preferences for infant-directed singing. *Infant Behav. Dev.* **20**, 383–396 (1997).
17. T. Nakata, S. E. Trehub, Expressive timing and dynamics in infant-directed and non-infant-directed singing. *Psychomusicology Music. Mind Brain* **21**, 45–53 (2011).
18. A. D. Patel, J. R. Iversen, J. C. Rosenberg, Comparing the rhythm and melody of speech and music: The case of British English and French. *Acoust. Soc. Am.* **19**, 3034–3047 (2006).
19. G. E. Peterson, I. Lehiste, Duration of Syllable Nuclei in English. *J. Acoust. Soc. Am.* **32**, 693–703 (1960).
20. P. Boersma, D. Weenink, Praat: doing phonetics by computer [Computer program] (2016).
21. O. Lartillot, “MIRtoolbox 1.7 User’ s Manual” (2017).
22. A. Fernald, P. Kuhl, Acoustic determinants of infant preference for motherese speech. *Infant Behav. Dev.* **10**, 279–293 (1987).
23. S. E. Trehub, J. Plantinga, F. A. Russo, Maternal Vocal Interactions with Infants: Reciprocal Visual Influences. *Soc. Dev.* **25**, 665–683 (2016).
24. L. K. Cirelli, Z. B. Jurewicz, S. E. Trehub, Effects of Maternal Singing Style on Mother–Infant Arousal and Behavior. *J. Cogn. Neurosci.*, 1–8 (2019).
25. F. De la Torre, *et al.*, Facial expression analysis. *IEEE Int. Conf. Autom. Face Gesture Recognit.* (2015).
26. X. Xiong, F. De La Torre, Supervised descent method and its applications to face alignment in *Proceedings of the IEEE Computer Society Conference on Computer Vision and Pattern Recognition*, (2013) https:/doi.org/10.1109/CVPR.2013.75.

27. D. N. Stern, “Mother and infant at play: The dyadic interaction involving facial, vocal, and gaze behaviors.” in *The Effect of the Infant on Its Caregivers*, M. Lewis, L. A. Rosenblum, Eds. (Wiley-Interscience, 1974), pp. 187–213.
28. S. C. F. Chong, J. F. Werker, J. A. Russell, J. M. Carroll, Infant and Child Development Three Facial Expressions Mothers Direct to Their Infants. *Child Dev* **12**, 211–232 (2003).
29. D. N. Stern, *The First Relationship: Infant and Mother* (Harvard University Press, 1977).
30. L. B. Adamson, *Communication Development During Infancy* (Routledge, 2018).
31. P. Ekman, H. Oster, Facial expressions of emotion. *Annu. Rev. Psychol.* **30**, 527–554 (1979).
32. J. H. Zar, *Biostatistical Analysis*, 5th Ed. (Prentice-Hall/Pearson, 2010).
33. P. Berens, CircStat : A MATLAB Toolbox for Circular Statistics . *J. Stat. Softw.* **31**, 1–21 (2009).
34. S. R. Jammalamadaka, A. SenGupta, *Topics in Circular Statistics* (WORLD SCIENTIFIC, 2001).
35. J. Tchorz, B. Kollmeier, Estimation of the signal-to-noise ratio with amplitude modulation spectrograms. *Speech Commun.* **38**, 1–17 (2002).
36. A. Fernald, T. Simon, Expanded intonation contours in mothers’ speech to newborns. *Dev. Psychol.* **20**, 104–113 (1984).
37. L. Trainor, C. Zacharias, Infants prefer higher-pitched singing. *Infant Behav. Dev.* **21**, 799–805 (1998).
38. C. D. Tsang, S. Falk, A. Hessel, Infants Prefer Infant-Directed Song Over Speech. *Child Dev.* **88**, 1207–1215 (2017).
39. S. Falk, N. Audibert, Acoustic signatures of communicative dimensions in codified mother-infant interactions. *J. Acoust. Soc. Am.* **150**, 4429–4437 (2021).
40. A. Fernald, C. Mazzie, Prosody and focus in speech to infants and adults. *Dev. Psychol.* **27**, 209–221 (1991).
41. J. Colombo, J. Frick, J. Ryther, J. Coldren, D. Mitchell, Infants’ detection of analogs of" motherese" in noise. *Merrill. Palmer. Q.* **41**, 104–113 (1995).
42. S. R. Livingstone, W. F. Thompson, M. M. Wanderley, C. Palmer, Common cues to emotion in the dynamic facial expressions of speech and song. *Q. J. Exp. Psychol.* **68**, 952–970 (2015).
43. E. B. Lange, J. Fünderich, H. Grimm, Multisensory integration of musical emotion perception in singing. *Psychol. Res.* (2022) https:/doi.org/10.1007/S00426-021-01637-9.
44. E. Bigand, Perceiving musical stability: the effect of tonal structure, rhythm, and musical expertise. *J. Exp. Psychol. Hum. Percept. Perform.* **23**, 808–822 (1997).
45. E. Bigand, M. Pineau, Context effects on melody recognition: A dynamic interpretation. *Curr. Psychol. Cogn.* **15**, 121–134 (1996).
46. M. R. Jones, M. Boltz, Dynamic Attending and Responses to Time. *Psychol. Rev.* **96**, 459–491 (1989).
47. M. R. Jones, “Dynamics of musical patterns: How do melody and rhythm fit together?” in *Psychology and Music: The Understanding of Melody and Rhythm*, 1st Ed., W. J. Dowling, T. J. Tighe, Eds. (Lawrence Erlbaum and Associates, Inc., 1993), pp. 67–92.
48. D. J. Lewkowicz, Learning and Discrimination of Audiovisual Events in Human Infants: The Hierarchical Relation Between Intersensory Temporal Synchrony and Rhythmic Pattern Cues. *Dev. Psychol.* **39**, 795–804 (2003).
49. L. E. Bahrick, R. Lickliter, Intersensory redundancy guides attentional selectivity and perceptual learning in infancy. *Dev. Psychol.* **36**, 190–201 (2000).
50. A. Antonietti, D. Cocomazzi, P. Iannello, Looking at the Audience Improves Music Appreciation. *J. Nonverbal Behav.* **33**, 89–106 (2009).
51. E. Coutinho, K. Scherer, The effect of context and audio-visual modality on emotions elicited by a musical performance. *Psychol. Music* **45**, 550–569 (2017).
52. S. Livingstone, W. Thompson, F. Russo, Facial expressions and emotional singing: A study of perception and production with motion capture and electromyography. *Music Percept.* **26**, 475–488 (2009).
53. W. F. Thompson, P. Graham, F. A. Russo, Seeing music performance: Visual influences on perception and experience. *Semiotica* **156**, 203–227 (2005).
54. D. K. Ceaser, W. F. Thompson, F. Russo, Expressing tonal closure in music performance: auditory and visual cues. *Can. Acoust. - Acoust. Can.* **37**, 29–34 (2009).
55. D. Huron, S. Dahl, R. Johnson, Facial expression and vocal pitch height: Evidence of an intermodal association. *Empir. Musicol. Rev.* **4**, 93–100 (2009).

56. W. F. Thompson, F. A. Russo, Facing the Music. *Psychol. Sci.* **18**, 756–757 (2007).
57. S. R. Livingstone, C. Palmer, Head movements encode emotions during speech and song. *Emotion* **16**, 365–380 (2016).
58. J. Davidson, A. Coulam, "Exploring jazz and classical solo singing performance behaviours: A preliminary step towards understanding performer creativity" in *Musical Creativity*, (Psychology Press, 2006), pp. 197–215.
59. J. W. Davidson, The Activity and Artistry of Solo Vocal Performance: Insights from Investigative Observations and Interviews with Western Classical Singers. *Music. Sci.* **11**, 109–140 (2007).
